# Functional connectotomy of a whole-brain model reveals tumor-induced alterations to neuronal dynamics in glioma patients

**DOI:** 10.1162/netn_a_00426

**Published:** 2025-03-20

**Authors:** Christoffer G. Alexandersen, Linda Douw, Mona L. M. Zimmermann, Christian Bick, Alain Goriely

**Affiliations:** Mathematical Institute, University of Oxford, Oxford, UK; Department of Anatomy & Neurosciences, Amsterdam Neuroscience, Cancer Center Amsterdam, Amsterdam UMC, Amsterdam, The Netherlands; Department of Mathematics, Vrije Universiteit Amsterdam, Amsterdam, The Netherlands; Amsterdam Neuroscience – Systems & Network Neuroscience, Amsterdam, The Netherlands

**Keywords:** Functional connectivity, Whole-brain model, MEG, Glioma, Neuronal dynamics, Neural oscillations, Networks, Dynamical systems

## Abstract

Brain tumors can induce pathological changes in neuronal dynamics that are reflected in functional connectivity measures. Here, we use a whole-brain modeling approach to investigate pathological alterations to neuronal activity in glioma patients. By fitting a Hopf whole-brain model to empirical functional connectivity, we investigate glioma-induced changes in optimal model parameters. We observe considerable differences in neuronal dynamics between glioma patients and healthy controls, both on an individual and population-based level. In particular, model parameter estimation suggests that local tumor pathology causes changes in brain dynamics by increasing the influence of interregional interactions on global neuronal activity. Our approach demonstrates that whole-brain models provide valuable insights for understanding glioma-associated alterations in functional connectivity.

## Author Summary

This study investigates how gliomas affect neuronal activity and connectivity using a whole-brain computational model. By fitting this model to empirical data, we compare glioma patients with healthy individuals to uncover significant differences in brain dynamics. Our findings indicate that local tumor pathology enhances the influence of interregional interactions on overall neuronal activity. This approach underscores the utility of whole-brain computational models in revealing the complex alterations in functional connectivity associated with gliomas, advancing our understanding of their impact on brain function.

## INTRODUCTION

One of the ultimate goals of neuroscience is to understand and control neuronal dynamics across scales, from the individual neuron to the whole brain. At the larger end of the scale, researchers have focused on imaging methods such as functional magnetic resonance imaging (fMRI), electroencephalography (EEG), and magnetoencephalography (MEG) to measure whole-brain dynamics. These methods have revealed consistent correlation patterns in neuronal activity across brain regions. Interestingly, these correlation patterns, commonly known as functional connectivity, are disrupted in diseases such as Alzheimer’s disease (Jalilianhasanpour, Beheshtian, Sherbaf, Sahraian, & Sair, 2019), depression (X.-Y. Li, Chen, & Yan, 2022), schizophrenia (S. Li et al., 2019), and glioma (Derks, Reijneveld, & Douw, 2014; Fox & King, 2018; Maas & Douw, 2023). Following these observations, computational neuroscientists have been formulating *whole-brain models* to explain functional connectivity patterns and how they change during disease. However, fitting the model output to functional connectivity is a significant challenge, even for simple brain states. Overcoming this challenge is crucial, as it is a major roadblock for using whole-brain models in clinical applications, which has the potential to improve diagnosis, to understand disease mechanisms, and to enable personalized treatments (Deco & Kringelbach, 2014; Lang et al., 2023; Pathak, Roy, & Banerjee, 2022). For example, patients with brain tumors such as gliomas exhibit pathological alterations in functional connectivity that are not yet fully understood; the development of accurate whole-brain models can help reveal the underlying mechanisms of tumor pathology. Here, we employ a whole-brain model to elucidate the mechanisms that lead to functional connectivity alterations in glioma patients and determine the impact tumor regions have on the emergent whole-brain dynamics.

It is now well established that glioma patients have been shown to exhibit seemingly pathological differences in functional connectivity patterns across imaging modalities when compared with healthy controls (Derks et al., 2014; Fox & King, 2018; Maas & Douw, 2023). In particular, overall functional connectivity has been shown to be lower in the default-mode network as measured by fMRI (Esposito et al., 2012; Ghumman, Fortin, Noel-Lamy, Cunnane, & Whittingstall, 2016; Harris et al., 2014; Maesawa et al., 2015). Similarly, lower functional connectivity is observed in MEG for higher frequency bands (Bartolomei et al., 2006a, 2006b; Bosma et al., 2008; Guggisberg et al., 2008), whereas lower frequency bands quite consistently display higher functional connectivity compared with controls instead (Bartolomei et al., 2006a; Bosma et al., 2008, 2009). Further differences in functional connectivity have been observed in network metrics of MEG/fMRI functional connectivity such as clustering and path length (Bartolomei et al., 2006a; Bosma et al., 2009; van Dellen et al., 2012; Xu et al., 2013). However, it remains unclear why and exactly how such connectivity deviations occur in some patients, but not all (Derks et al., 2021).

Whole-brain models are formulated to simulate empirical functional connectivity patterns and have been used recently to study a variety of diseases. Essentially, a whole-brain model is a network of coupled oscillators in which each oscillator produces a time series mimicking the oscillatory signals of a region found through neuroimaging modalities. These simulated signals can then be used to compute simulated functional connectivity, which is then compared with experimental functional connectivity. The basic network architecture of a whole-brain model comes from structural reconstructions of the human brain, called structural connectomes. In a structural connectome, the network’s nodes denote brain regions of various sizes and the network’s edges encode some measure of physical connectivity between the regions. The most common measure is the integrity of axonal bundles divided by their length as reconstructed from diffusion magnetic resonance imaging (dMRI). The dynamical model of neuronal activity at each node varies from simple phenomenological oscillators to high-dimensional biophysical models. Whole-brain models have long been used to replicate resting-state functional connectivity as measured by fMRI, EEG, and MEG (Cabral, Hugues, Sporns, & Deco, 2011; Deco, Jirsa, & McIntosh, 2011; Freyer et al., 2011). Additionally, whole-brain models have been used to investigate brain diseases and neuropsychiatric disorders, such as epilepsy (Jirsa et al., 2017; Taylor, Kaiser, & Dauwels, 2014), Alzheimer’s disease (Alexandersen, de Haan, Bick, & Goriely, 2023; Demirtaş et al., 2017; Goriely, Kuhl, & Bick, 2020; Stefanovski et al., 2019), schizophrenia (Anticevic et al., 2012; Cabral et al., 2013; Yang et al., 2014), and glioma (Aerts, Fias, Caeyenberghs, & Marinazzo, 2016; Aerts et al., 2018, 2020; Alstott, Breakspear, Hagmann, Cammoun, & Sporns, 2009).

Whole-brain models can be used to simulate the effect of lesions to better understand the effect of surgical craniotomy, which often is necessary for epilepsy and glioma patients (Alstott et al., 2009). In such studies, a set of brain regions is removed from the physical brain network structure of the whole-brain model (virtual surgery), and the resulting changes in functional connectivity are analyzed. The effect of brain region removal on the emergent functional connectivity patterns is nontrivial and depends on the structural connectivity network (Aerts et al., 2016; Nissen et al., 2021). For example, the effect of removing certain regions on fMRI-derived functional connectivity depends largely on the region. The removal of some regions—such as those in the cortical midline and the temporoparietal junction—results in global changes, whereas other regions—such as primary sensory and motor regions—result only in local changes (Alstott et al., 2009). Another highlight is the resilience of brain dynamics to surgical removal and the importance of node network measures in determining the impact of lesions (Aerts et al., 2016). Furthermore, differences in neuronal dynamics can be inferred by optimizing the parameters of the whole-brain model to glioma patients and control subjects. For example, using fMRI-derived functional connectivity and a reduced Wong-Wang model for neuronal dynamics, local inhibitory connections were considerably lower in tumor regions, whereas no significant difference was found in long-range, interregional connections (Aerts et al., 2018). Moreover, optimal parameter fits of the (reduced Wong-Wang) whole-brain model remain relatively stable after comparing before and after real-world craniotomy, and the capability of whole-brain models to accurately predict the effects of craniotomy in glioma patients is, as of yet, difficult to ascertain due to the small cohort sizes of glioma patients currently available (Aerts et al., 2020). Furthermore, considering changes induced by glioma in EEG/MEG, a whole-brain model approach demonstrated that tumor-induced changes in local neuronal dynamics can considerably reduce the overall functional connectivity strength, suggesting that local pathology can lead to global symptoms (van Dellen et al., 2013).

In this study, we use a whole-brain model of Hopf oscillators to explain the functional connectivity differences in glioma patients as compared with healthy controls, measured by MEG. We do so by identifying the optimal parameterization of the whole-brain model to empirical phase-lag index (PLI) connectivity computed from MEG data of glioma patients and healthy controls. We find that the goodness of fit of the Hopf whole-brain model is invariant under a particular scaling law for the parameters. This scaling law demonstrates that the phase dynamics is almost solely determined by the *normalized coupling strength*, which we define as the ratio of interregional coupling and intraregional excitability. Furthermore, we find that the optimal normalized coupling strength is higher in the glioma cohort than in the controls both on an individual and population-based level. We also show that the tumor regions generally induce increases in normalized coupling strength, though there is considerable variability between patients. In summary, we find that the ratio of interregional and intraregional dynamics uniquely determines the phase dynamics of the Hopf whole-brain model and that tumors increase the relative contribution of interregional dynamics in glioma patients.

## METHODS

The outline of the paper is shown in Figure 2. A whole-brain model using a structural connectome averaged over the healthy cohort is used to simulate MEG-based functional connectivity using the PLI metric. Optimal model parameters are found using a grid search over a sample of random initial conditions (see Figure 2A). The fitting procedure is repeated for the average healthy cohort, average glioma cohort, and individual glioma patients. The distribution of optimal parameters over initial conditions is then compared between the patients and the control (see Figure 2B). Finally, functional connectotomy is performed for each individual patient, in which the tumor-related brain regions are removed from the empirical and simulated functional connectivity matrices, and the optimal model parameters are recomputed. The change in optimal model parameters induced by functional connectotomy is then compared with controls to identify how the tumor regions impact the whole-brain dynamics of the computational model (see Figure 2C).

### Participants

A total of *S* = 10 patients seen between 2011 and 2023 at Amsterdam UMC with suspected diffuse glioma were randomly selected from an ongoing prospective study on brain networks. Patients underwent MEG when glioma was suspected based on clinical history and MRI before tumor treatment or surgery was performed. Exclusion criteria were (a) age < 18 years, (b) psychiatric disease, (c) comorbidities of the central nervous system, (d) insufficient mastery of the Dutch language, and (e) inability to communicate adequately. Additionally, *S* = 33 healthy controls were included, which are further described in Breedt et al. (2023). We refer to these two cohorts as the *glioma cohort* and the *control cohort*. The VUmc Medical Ethical Committee approved this study, which was conducted following the principles of the Declaration of Helsinki. All participants provided written informed consent before participation.

### MEG Data Acquisition and Processing

MEG was recorded for 5 min in supine position during an eyes-closed, no-task resting state in a magnetically shielded room (VacuumSchmelze GmBh, Hanau, Germany), using a 306-channel (102 magnetometers, 204 gradiometers), whole-head MEG system (Elekta Neuromag Oy, Helsinki, Finland) and a sampling frequency of 1250 Hz. Anti-aliasing (410 Hz) and high-pass filters (0.1 Hz) were applied online. Preprocessing involved visual inspection, noisy channel removal, and noise removal in the remaining signals. Anatomical MRI was used for coregistration with the digitized scalp surface and the automated anatomical labeling atlas (Tzourio-Mazoyer et al., 2002) for parcellation of the cortical ribbon into 78 regions. Broadband time series of neuronal activity were then reconstructed for each region’s centroid (Hillebrand et al., 2016) using a scalar beamformer approach (Hillebrand, Barnes, Bosboom, Berendse, & Stam, 2012). Epochs of 13.5 s were then curated for each subject, which was then subsequently used to compute PLI as outlined below.

Median peak frequencies of the spectral density were also computed for the control cohort (for the parameterization of the whole-brain model). For this purpose, epochs were curated visually by discarding epochs without clear alpha frequency peaks in occipital brain regions. Then, the peak frequency of each region of interest in each epoch was computed by finding the frequency above 4 Hz with the highest spectral density and discarding peaks that were less than twice the average spectral density of the background spectrum. The median peak frequency of each brain region was then computed across all subjects and epochs.

### MRI Data and Structural Connectome Reconstruction

Control MRI data were obtained using a 3 T MRI system (Philips Ingenia CX) with a 32-channel, receive-only head coil at the Spinoza Centre for Neuroimaging in Amsterdam, The Netherlands. A high-resolution 3D T1-weighted image was collected with a magnetization-prepared rapid acquisition with gradient echo (MPRAGE; TR = 8.1 ms, TE = 3.7 ms, flip angle = 8°, voxel dimensions = 1 mm^3^ isotropic). This anatomical scan was registered to MNI space through linear registration with a nearest-neighbor interpolation and was used for coregistration and normalization of other modalities (dMRI and MEG) to the same space.

Diffusion MRI was collected in the controls only, with diffusion weightings of *b* = 1,000 and 2,000 s/mm^2^ applied in 29 and 59 directions, respectively, along with nine non-diffusion-weighted (*b* = 0s/mm^2^) volumes using a multiband sequence (MultiBand SENSE factor = 2, TR = 4.7 s, TE = 95 ms, flip angle = 90°, voxel dimensions = 2 mm^3^ isotropic, no interslice gap). In addition, two scans with opposite phase encoding directions were collected for blip-up blip-down distortion correction using an FSL topup (Andersson, Skare, & Ashburner, 2003). Structural connectomes were constructed by performing probabilistic anatomically constrained tractography (ACT; Smith, Tournier, Calamante, & Connelly, 2012) in MRtrix3 (Tournier et al., 2019). A tissue response function was estimated from the preprocessed and bias field-corrected dMRI data using the multishell, multitissue, five-tissue-type algorithm (msmt_5tt). Subsequently, the fiber orientation distribution for each voxel was determined by performing a multishell, multitissue-constrained spherical deconvolution (MSMT-CSD; Jeurissen, Tournier, Dhollander, Connelly, & Sijbers, 2014). ACT was performed by randomly seeding 100 million fibers within the white matter to construct a tractogram, and spherical-deconvolution informed filtering of tractograms (SIFT, SIFT2 method in MRtrix3; Smith, Tournier, Calamante, & Connelly, 2015) was then performed to improve the accuracy of the reconstructed streamlines and reduce false positives. For every participant, their respective 3D T1-weighted image was used to parcellate the brain into the 78 cortical regions. We then used this parcellation to convert the tractogram to a structural network, where weighted edges represented the sum of all streamlines leading to and from all voxels within two brain regions. For patients, tumor masks were manually drawn on a combination of T1-weighted MRI with and without contrast, and FLAIR. Then, the atlas regions overlapping with each patient’s tumor mask were considered tumor regions.

### Computation of the PLI as a Measure of Functional Connectivity

In this study, we used the PLI as a measure of functional connectivity (Stam, Nolte, & Daffertshofer, 2007). PLI is a measure of the average asymmetry in pairwise phase differences of oscillatory MEG signals from different brain regions and ranges from 0 (symmetry) to 1 (asymmetry) for each pair of regions.

For each epoch, the recorded MEG signal was first bandpassed using a Butterworth filter into the alpha range (8–12 Hz). Then the angle of the filtered signal was computed using the Hilbert transform and subsequently used to compute the PLI signal. The PLI of each subject was computed as the average PLI over their epochs.

The experimental PLI matrices are noisy and are thus thresholded to ease the comparison with the noiseless whole-brain model. Thresholding the PLI matrix at a certain percentage *X*% means setting all elements in the lower *X*% percent to zero as ordered per magnitude. For further details on the PLI computation, see the Supporting Information.

### The Hopf Whole-Brain Model

We use a whole-brain model consisting of a network of Hopf oscillators. More specifically, we use the Hopf normal form (equivalent to Stuart-Landau oscillators) on a reconstruction of the physical human brain network, where the oscillators interact through a sigmoidal function without delays. As such, each Hopf oscillator denotes a brain region, where the links of the network denote axonal bundles connecting a pair of brain regions.

The human structural connectome network is represented by the adjacency matrix *W* with entries *w*_*ij*_. The state of the Hopf oscillators, *z*_*i*_ ∈ ℂ, are complex variables having real and complex parts *z*_*i*_ = *x*_*i*_ + i*y*_*i*_, where i = $\sqrt{-1}$. The Hopf oscillators are coupled through their real parts *x*_*i*_, and a subsequent analysis of simulations will only consider the real part and not the imaginary part. Indeed, the real part *x*_*i*_ represents the simulated MEG signals, while the imaginary part *y*_*i*_ can be thought of as a hidden variable needed to produce the necessary oscillatory dynamics.

The Hopf whole-brain model for a network *W* with *N* regions evolves through the following system of complex ordinary differential equations

$${\dot{z}}_{i}=F\left(z_{i};\lambda ,\omega_{i}\right)+K\tanh\left(C\sum_{j=1}^{N}w_{ij}x_{j}\right),\;i=1,\ldots ,N,$$ (1)

with

$$F\left(z_{i};\lambda ,\omega_{i}\right)=z_{i}\left(\lambda +\text{i}\omega_{i}-|z_{i}^{2}|\right).$$ (2)

Here, *K* ∈ ℝ^+^ = {*r* ∈ ℝ | *r* ≥ 0} is the *global coupling strength*, λ ∈ ℝ is the *global excitability parameter* (also known as the Hopf bifurcation parameter), *ω*_*i*_ ∈ ℝ^+^ is the local natural frequency of oscillation, and *C* ∈ ℝ^+^ is the *global scaling* of the structural connectome.

Each natural frequency *ω*_*i*_ is parameterized from the healthy control MEG data using the frequency peaks found for each brain region. Thus, the remaining free parameters are the coupling strength *K*, the excitability *λ*, and the structural connectivity scaling *C*.

### Fitting Whole-Brain Model to Experimental Functional Connectivity

To assess the goodness of fit of the Hopf whole-brain model to experimental MEG data, we compare the functional connectivity of the simulated and experimental signals. The objective (cost) function we use to assess the goodness of fit is the Pearson correlation of the time-averaged pairwise PLIs.

We simulate the Hopf whole-brain model for 14.5 s and discard the first second of simulations to account for transient dynamics induced by the initial conditions. We save the simulated data with a sampling frequency of 1250 Hz. As such, the simulated data have the same temporal length (13.5 s) and sampling frequency as the experimental MEG data. The simulations are performed with the RK45 method (using the JiTCODE module for Python) with an absolute tolerance of 10^−6^ and a relative tolerance of 10^−3^. The angle of the filtered simulated data (as computed by the Hilbert transform and a Butterworth filter for 8–12 Hz) is then used to compute a simulated PLI. We determine the goodness of fit of the model by the Pearson correlation between the simulated and empirical PLI data. In the Results section, we investigate the impact of tumor regions on the dynamics of the whole-brain model in individual patients and clusters of tumor regions. We do so by performing a *functional connectotomy*, in which we remove the rows and columns corresponding to the tumor brain regions from both the experimental and simulated PLI matrices. In effect, the objective function ignores the tumor regions, resulting in a change in optimal model parameters. The settings of the whole-brain model simulations, the processing of the simulated data, and the computation of the objective function are further described in the Supporting Information.

### Statistical Tests for Changes in Coupling Strength

As we fit the Hopf whole-brain model to the empirical functional connectivity of healthy controls and glioma patients, we want to determine whether the optimal model parameters are different between the groups. Moreover, we will compute optimal model parameters for a large number of initial conditions of the computational model, giving us—for each empirical fit—distributions of optimal parameters over the initial conditions. We perform double-sided Wilcoxon rank-sum tests to determine whether these distributions (e.g., one being the control and another being a patient) have the same arithmetic mean. For the functional connectotomy, we first—for both the control and patients—compute the optimal model parameters before and after the functional connectotomy. We then have distributions of changes in optimal model parameters due to functional connectotomy. We then test whether the means of these distributions are the same for the patients and the control using the Wilcoxon rank-sum test. We perform these tests with the Scipy package from Python (Virtanen et al., 2020).

## RESULTS

### Phase Correlations Emerge From the Competition Between Inter- and Intraregional Dynamics

**Figure 1.**
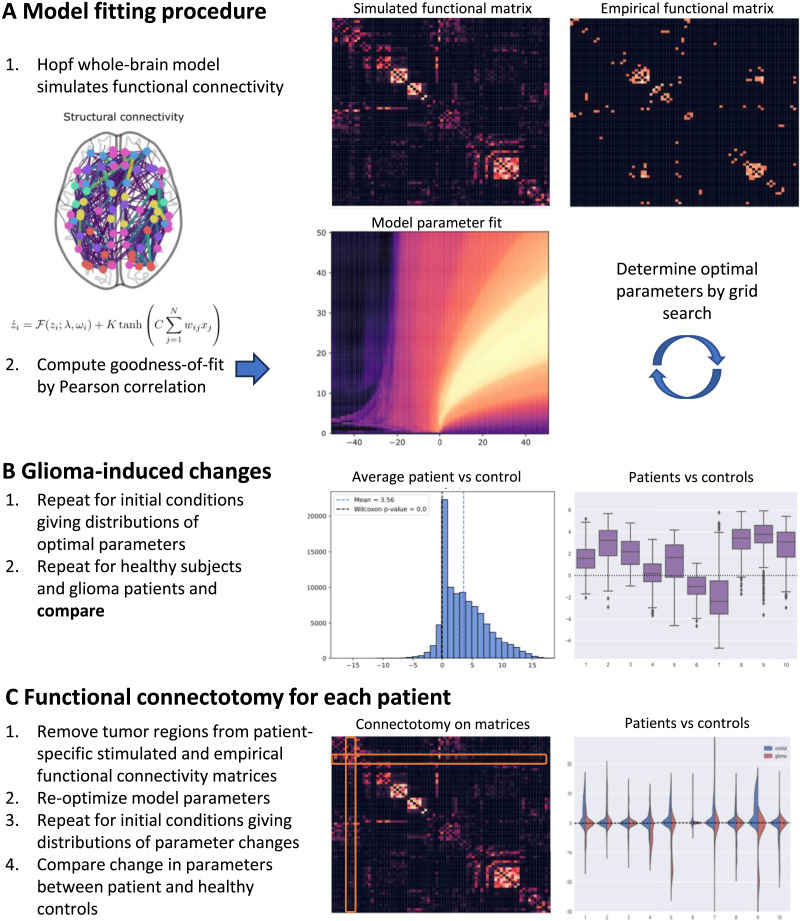
Overview of the model fitting and functional connectotomy. (A) The Hopf whole-brain model simulates the E/MEG signal, which can be used to produce simulated functional connectivity matrices. These are then compared with empirical functional connectivity to find optimal model parameters. (B) We determine optimal model parameters over a large number of initial conditions of the whole-brain model. We repeat this for both patients and average healthy control to determine glioma-induced changes in brain dynamics. (C) To determine the contribution of tumor regions, we perform a functional connectotomy where tumor regions are removed from the functional connectivity. We then re-optimize the model, finding new optimal parameters, both for patients and the control. The changes in optimal parameters reflect the contribution of the tumor regions to the whole-brain dynamics.

**Figure 2.**
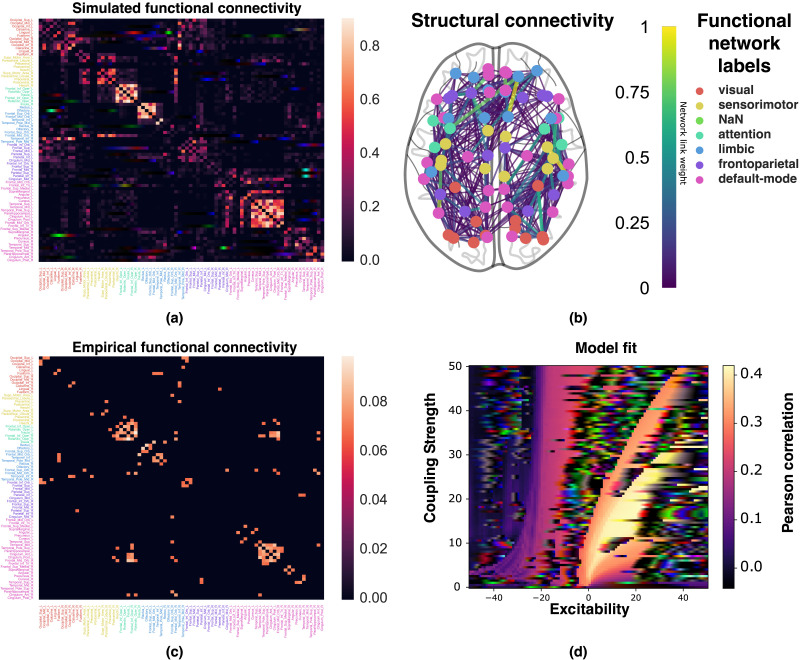
(A and C) An example of a synthetic (simulated) PLI from a Hopf whole-brain model and experimental PLI averaged over 33 healthy subjects from the control group. The name of each brain region is labeled and colored according to canonical functional networks (see legend to the upper right). (B) The weighted physical connectome averaged over the control group. Each node is colored similarly to the functional connectivity matrices. (D) The goodness of fit of the Hopf whole-brain model with respect to the coupling strength and excitability parameter. Each pixel shows the Pearson correlation between the simulated and experimental (control group average) PLIs averaged over 500 whole-brain simulations with random initial conditions.

We compare simulations with experimental data to assess the goodness of fit for the Hopf whole-brain model (see Figure 1A for an overview). Specifically, we compute the PLI of the simulated signals of the whole-brain model (example shown in Figure 2A) and compare them with the averaged experimental PLI of a healthy control group (experimental PLI found in Figure 2C). The goodness of fit is defined as the Pearson correlation between the simulated and experimental pairwise PLIs.

The experimental PLI is noisy and is thus thresholded to make it comparable with the noiseless, simulated PLI. Figure S3 in the Supporting Information shows the effect of thresholding on the average control PLI. We threshold the experimental, averaged PLI at the optimal threshold, providing the best fit to the simulated data (see Supporting Information Figures S4 and S5). However, we note that the following results hold for a wide range of threshold values, as demonstrated in the Supporting Information. In particular, the results have also been reproduced by thresholding the experimental PLI by their median value so that all elements of the PLI matrix below the median value are set to zero (see Supporting Information Figure S6).

The structural connectivity used to provide the network of the Hopf whole-brain network is the averaged dMRI connectome of the healthy control cohort (see Figure 2B). The structural connectivity is unitless, and hence, we use a scaling constant *C* as a free parameter. We found that the goodness of fit of the Hopf whole-brain model is not sensitive to structural connectivity scaling (see Supporting Information Figure S1). As long as the scaling parameter is large enough, it has no impact on the goodness of fit. As such, we parameterize the scaling at *C* = 20 and keep it this way for the remainder of the study. The natural frequencies of the individual brain regions must also be parameterized in the Hopf whole-brain model. We set the natural frequencies equal to the average peak frequencies of the experimental MEG signal of the healthy cohort. As such, the only remaining parameters of the Hopf whole-brain model are the global coupling strength *K* and the global excitability parameter *λ*.

We perform a two-dimensional grid search over the coupling strength and excitability parameter to find the optimal parameterization of the Hopf whole-brain model to average control PLI connectivity as shown in Figure 2D. Contrary to previous modeling assumptions, we find that the optimal fit to functional connectivity occurs at positive excitability parameter values (*λ* > 0). We also see that there are contour lines over the coupling strength and positive excitability parameters where the goodness of fit does not change. This suggests that the phase dynamics (as measured by PLI) are invariant—to some degree—under some transformation for positive excitability values.

**Figure 3.**
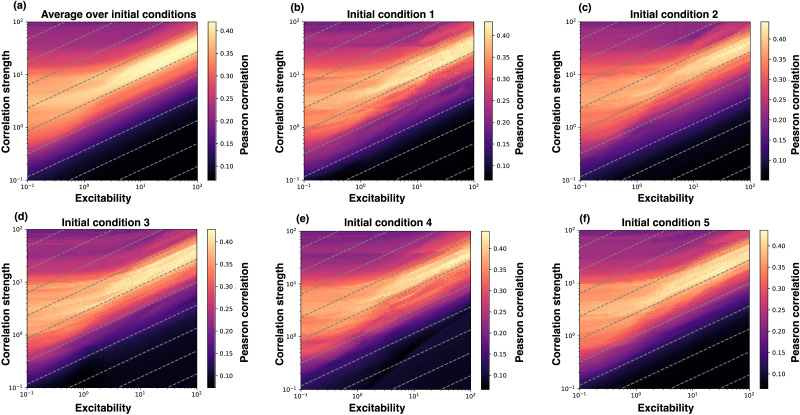
Parameter grid-search on logarithmic axes for a Hopf whole-brain model for individual initial conditions (B–F) and averaged over 300 initial conditions (A). The gray stippled lines represent a square-root scaling of the coupling strength with respect to excitability. That is, the gray stippled lines show $K=k\sqrt{\lambda }$ for different *k*. Each pixel shows the Pearson correlation between the simulated and experimental (average, control group) PLI connectivity. The grids indicate that the Pearson correlation is unchanged as the coupling strength scales with the square root of excitability, such that $K∼\sqrt{\lambda }$.

Rescaling the variables of the Hopf whole-brain model by ${\tilde{x}}_{i}=\sqrt{\lambda }x_{i},{\tilde{y}}_{i}=\sqrt{\lambda }y_{i}$ reveals a modified coupling constant $K/\sqrt{\lambda }$ (see the Supporting Information). As such, we expect the phase dynamics of the Hopf whole-brain model to be largely determined by the ratio $K/\sqrt{\lambda }$ and not by the coupling strength and excitability independently. We see that this is indeed the case when computing the goodness of fit over coupling strength and excitability on a log–log grid (see Figure 3), showing that the goodness of fit is invariant under $K∼\sqrt{\lambda }$. The coupling strength is a measure of interregional (global) coupling, whereas the excitability parameter is a measure of intraregional (local) excitability. As such, it is the competition between interregional and intraregional dynamics that determines the phase correlations in the Hopf whole-brain model. We will refer to the ratio $k=K/\sqrt{\lambda }$ as the *normalized coupling strength*.

### Whole-Brain Modeling Reveals Higher Interregional Coupling in Glioma Cohort

**Figure 4.**
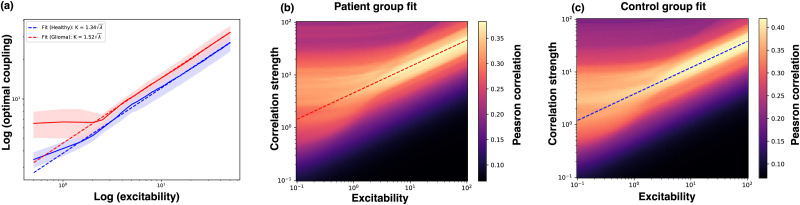
Optimal parameter fits of the Hopf whole-brain model to experimental PLI connectivity for the control (blue) and glioma (red) cohort average. The optimal normalized coupling strength is fitted by regression (stippled line) and is the slope of the average optimal coupling strength per excitability parameter (solid line). Notice that all plots are in log-log coordinates. (a) The average (line) and standard deviation (shaded region) of optimal excitability and coupling strength over 1,000 initial conditions. The average optimal coupling strength is fitted to a square-root dependence on the excitability (Hopf) parameter per the healthy (blue) and glioma (red) cohorts. The regression fits the average optimal parameters well. (B and C) The fitted line (regression) of the optimal ratio between coupling strength and excitability parameters is shown on the parameter grid search for the healthy and glioma cohorts.

We have now determined that it is the normalized coupling strength that controls the phase dynamics of the Hopf whole-brain model, and we can now investigate whether the normalized coupling strength differs between glioma patients and healthy controls (see Figure 1B for an overview). As before, we perform two-dimensional grid searches over the coupling and excitability parameters, computing the goodness of fit to the averaged PLI of the healthy control group and the glioma group, respectively (Figure 4B and 4C). We then find the optimal coupling strength per excitability parameter for both cohorts and compute the mean and standard deviation of the optimal coupling strength over randomized initial conditions for the whole-brain simulations (Figure 4A, solid lines).

We find that the glioma cohort has a higher coupling strength over the excitability parameters, suggesting that their *normalized coupling strength* is higher. To confirm this, we find the optimal fit for the mean optimal coupling strength per the square root of the excitability parameter through least squares regression. That is, we find the scaling *k* providing the best fit for $K=k\sqrt{\lambda }$ for the mean of the two cohorts. As shown in Figure 4A, the regression provides an excellent fit for larger excitability values and produces a higher normalized coupling strength for the glioma cohort compared with the control cohort. The fitted regression lines of the normalized coupling strength have been plotted on top of the two-dimensional grid searches in Figure 4B and 4C.

**Figure 5.**
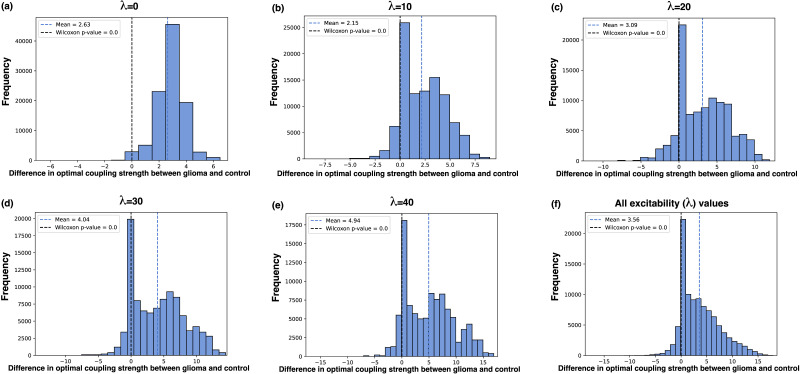
Distributions of the difference in optimal coupling strength between the healthy and glioma cohort for fixed excitability parameters over 1,000 initial conditions for the Hopf whole-brain model. The black stippled line illustrates where there is zero difference between the cohorts, and the blue stippled line shows the mean of the distributions. (A–E) For different excitability (Hopf) parameter values, we find the difference in optimal coupling strength between the averaged control and glioma cohort when optimized for the best fit to experimental PLI connectivity. The difference is positive when the glioma cohort has a higher optimal coupling strength and negative when the glioma cohort has a lower optimal coupling strength. The distributions arise from 1,000 simulations of the Hopf whole-brain model for random initial conditions. (F) In this histogram, all the optimal coupling strengths for all the excitability parameters (ranging from 0 to 50 with a step of 0.1) are pooled together into one larger distribution.

To further investigate the differences in functional connectivity between the control and glioma cohorts, we compute the distribution of optimal whole-brain parameters over the initial conditions for fixed excitability values. For 1,000 initial conditions, we compute the difference in optimal coupling strengths between the glioma and control cohorts for five different excitability parameter values and plot the resulting distributions in Figure 5A–E. Wilcoxon signed-rank tests were computed for the null hypothesis that the optimal parameter distributions (over initial conditions) have the same mean for the average control and average glioma patient. All tests showed highly significant results (*p* < 0.001) with a higher mean for the glioma average (λ = 0, Wilcoxon statistic = 6.40 × 10^5^, mean difference = 2.63; λ = 10, Wilcoxon statistic = 1.24 × 10^8^, mean difference = 2.15; λ = 20, Wilcoxon statistic = 1.85 × 10^8^, mean difference = 3.09; λ = 30, Wilcoxon statistic = 1.11 × 10^8^, mean difference = 4.04; λ = 40, Wilcoxon statistic = 9.73 × 10^7^, mean difference = 4.94; all λ pooled, Wilcoxon statistic = 1.37 × 10^8^, mean difference = 3.56). There is also considerable variation in the optimal coupling strength for different initial conditions. In particular, there seem to be two peaks in the distributions: one at zero difference and one at a positive difference. The bimodality disappears once all the optimal coupling strengths from the different excitability values are pooled into one distribution, as shown in Figure 5F. Many initial conditions do not exhibit different optima between the glioma and control cohorts. However, those that do exhibit differences have a bias toward higher coupling strength in the glioma cohort.

### Tumors Contribute to Pathological Interregional Coupling in Glioma Patients

**Figure 6.**
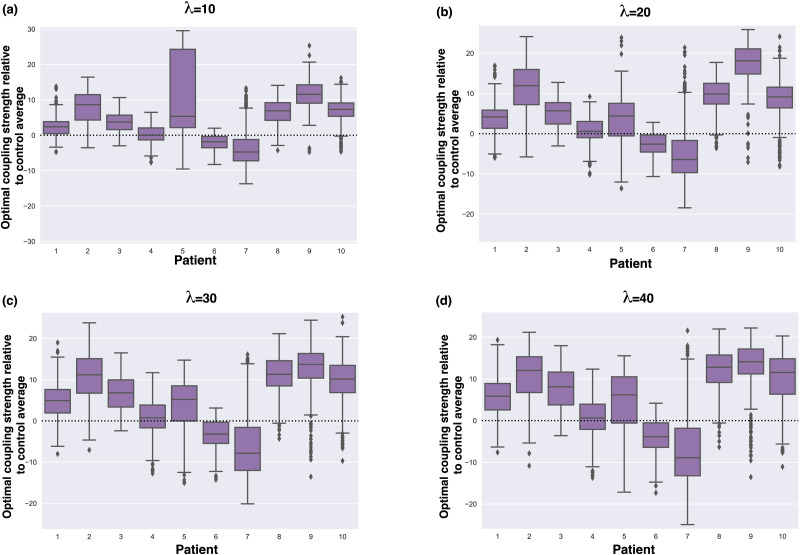
The difference in coupling strength found for individual patients and the healthy control, at different excitability parameter values λ. The box plots show the difference between optimal coupling strengths per patients’ individual PLI compared with the optimal coupling strength found per the healthy control, averaged over 500 initial conditions for the Hopf whole-brain model. A box distributed toward positive values indicates that the patients’ optimal coupling strength is higher than the optimal coupling strength for the healthy control average.

We have established that the normalized coupling strength (interregional vs. intraregional dynamics) is higher in the glioma cohort average when compared with controls. However, we will see that individual patients also exhibit higher normalized coupling strengths. For fixed excitability values, we find the optimal coupling strength when fitting simulated PLI to the *patient-specific* PLI (thresholded by their median value). We then compute the difference between these optimal coupling strengths to that of the average control group over different initial conditions, as shown in Figure 6. As demonstrated in the previous section, considerable variations between initial conditions can exist. Nevertheless, we observe a general trend in which patients individually have higher coupling strengths than expected. Wilcoxon signed-rank tests were computed for the null hypothesis that the optimal coupling strength distributions over initial conditions have the same mean for the patient as for the average control (see Table 1).

**Table 1.** Wilcoxon signed-rank test results for the functional connectotomy and patient-specific change in coupling strength

Patient	Functional connectotomy			Change in coupling strength		
	Statistic	*p* value	Mean difference	Statistic	*p* value	Mean difference
1	7.29 × 10^3^	<0.001	−4.8	4.84 × 10^3^	<0.001	1.6
2	2.31 × 10^4^	<0.001	1.7	1.20 × 10^3^	<0.001	2.9
3	2.15 × 10^4^	<0.001	1.1	5.86 × 10^2^	<0.001	2.1
4	2.11 × 10^4^	<0.001	−3.3	4.69 × 10^4^	<0.001	0.2
5	3.11 × 10^3^	<0.001	−8.2	2.44 × 10^4^	<0.001	1.1
6	2.13 × 10^3^	<0.001	−1.7	7.50 × 10^3^	<0.001	1.1
7	2.55 × 10^4^	<0.001	−0.3	2.09 × 10^4^	<0.001	−1.8
8	9.03 × 10^3^	<0.001	−3.3	2.23 × 10^2^	<0.001	3.25
9	2.59 × 10^2^	<0.001	−9.9	8.78 × 10^2^	<0.001	3.55
10	2.73 × 10^4^	0.003	0.2	3.10 × 10^3^	<0.001	2.7
cluster 1	2.69 × 10^4^	<0.001	−2.6
cluster 2	1.24 × 10^4^	<0.001	−3.5
cluster 3	3.87 × 10^4^	0.002	0.65
pooled pa.	1.82 × 10^6^	<0.001	−2.9
pooled cl.	3.76 × 10^5^	<0.001	−3.1

The null hypothesis for the functional connectotomy tests is that the change in optimal parameters after functional connectotomy has the same mean for the patient/cluster as for the control (see Figure 8C–F). The null hypothesis for the coupling strength change is that the distribution of optimal coupling strengths has the same mean for the patients and the control (see Figure 6).

To probe the role of tumors in shaping functional connectivity alterations, we perform a *functional connectotomy* in which we remove the tumor regions of the particular patient from the goodness-of-fit evaluation when finding optimal model parameters (see Figure 1C). The regions themselves are not removed from the model as such a virtual procedure would be too disruptive, but the objective function (Pearson correlation between simulated and empirical functional connectivity) ignores them when identifying optimal parameters. As such, if the optimal coupling strength is lower after functional connectotomy, it means that the tumors of that patient contribute to a pathologically high coupling strength.

**Figure 7.**
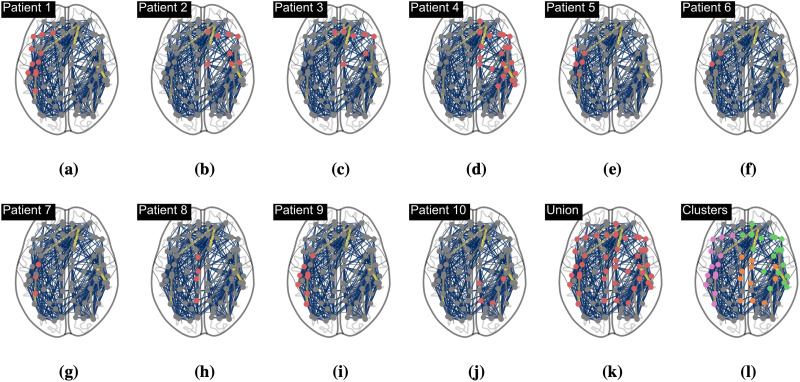
Functional connectotomies were performed to assess the contribution of tumors to the whole-brain model dynamics. Here, we show the locations of the tumor regions on top of the average structural connectivity. We also found three clusters (K-means clustering algorithm) of tumor regions, which will be used in subsequent analyses. The union of all tumor regions will also be used.

The tumor regions of the patients in the glioma cohort are shown in Figure 7A–J with their position in the physical structural connectome. For each patient, we first find the optimal parameters with respect to their complete, individual PLI connectome. We then recompute the optimal parameters with respect to their individual PLI connectome after functional connectotomy, that is, where the rows and columns of the tumor regions (all correlations involving the tumor regions) have been removed. As such, the change in optimal parameters reflects the impact the regions have on the goodness of fit of the whole-brain model and, by extension, their impact on the overall whole-brain dynamics.

We also identify three clusters of tumor regions by the K-means clustering algorithm, as shown in Figure 7K and 7F. In addition to investigating the tumor regions’ impact on global brain dynamics per patient, we also investigate the impact of the clusters—as well as the union of tumors across all patients—on the overall global dynamics, where we now fit the optimal parameters to the average PLI of the glioma cohort.

The optimal Pearson correlations across patients and average control (both with and without functional connectotomy) are shown in Supporting Information Figure S7. The Pearson correlation between simulated and empirical functional connectivity is higher than the correlation between structural connectivity and empirical functional connectivity, for all patients and the average control. Moreover, the Pearson correlation is also higher after functional connectotomy for all patients and the average control.

**Figure 8.**
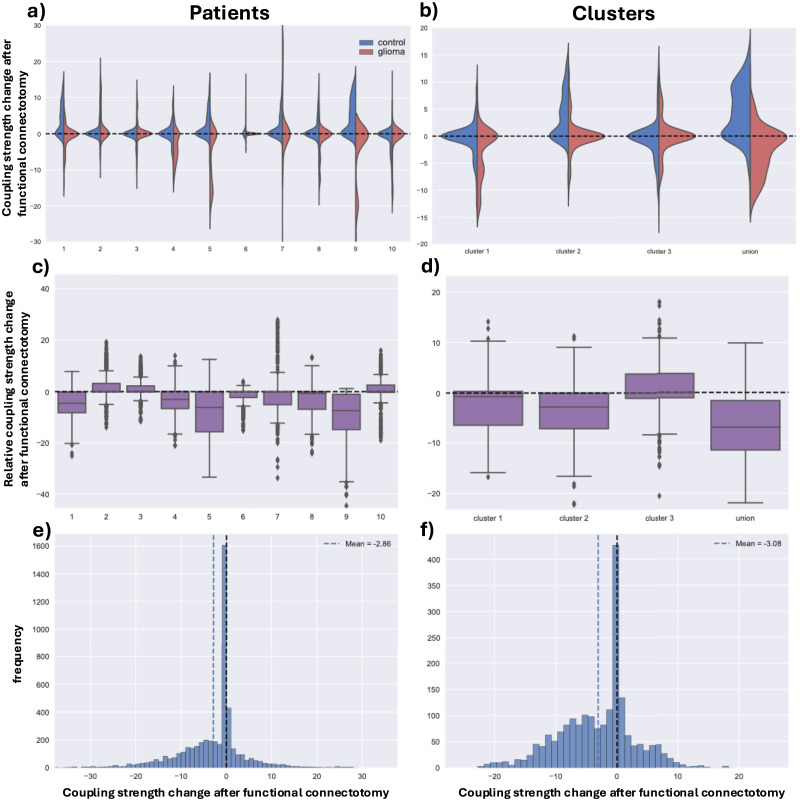
Functional connectotomies were performed to assess the impact of tumors on the Hopf whole-brain dynamics. Decreases in coupling strength after functional connectotomy indicate that the tumors are contributing to higher coupling strengths. For all simulations, the excitability parameter was kept constant (*λ* = 40) for over 500 initial conditions for the Hopf whole-brain model. Simulations were performed per patient (left column) and per tumor cluster (right column). (A and B) Violin plots of the change in optimal coupling strength after functional connectotomy. Optimal coupling strengths were found individually by computing the best fit to the patient’s phase-lag connectivity (red). We performed a control with the same functional connectotomy for each patient but fitted to the averaged healthy control (blue). (C and D) The difference in the change after functional connectotomy between the patients and healthy average control. If the box plots are centered toward negative values, then there are larger reductions in optimal coupling strength for the patient than compared with controls. (E and F) A histogram showing the distribution of changes in optimal coupling strength across all patients and initial conditions relative to the change in the average healthy control.

Setting the excitability parameter at *λ* = 40, we compute the optimal coupling strength before and after functional connectotomy for each patient and tumor cluster (see Figure 8). The goodness of fit is computed with patient-specific PLIs for each patient and averaged PLI (across patients) for each cluster. For each simulation, we perform the same patient-specific functional connectotomy to the average healthy control (fitting to the average healthy PLI). We use the average healthy structural connectome for both control and patient-specific simulations to keep the control and patient fit as comparable as possible. Moreover, previous studies found that subject-specific structural connectomes do not provide better fits to empirical functional connectivity than averaged connectomes (Aerts et al., 2018; Jirsa et al., 2017). Simulations are repeated over 500 initial conditions, with the resulting distributions shown in Figure 8A and 8C. There is considerable variation between patients, with some showing a clear reduction in coupling strength, whereas others are difficult to distinguish from their respective control. In Figure 8C and 8D, we show the distributions of the differences in the optimal coupling strength after functional connectotomy for each patient and their control. Wilcoxon signed-rank tests were computed for each patient/cluster/union with the null hypothesis that the change in optimal coupling strength after functional connectotomy has the same mean (sampled over initial conditions) between patients and control. All tests show highly significant results, with most patients and clusters showing lower coupling strengths than expected after functional connectotomy (see Table 1).

Patients show either no change or a decrease in coupling strength after functional connectotomy. We reiterate that decreases in coupling strength after functional connectotomy correspond to the tumors contributing to higher coupling strengths.

To further assess general changes caused by a functional connectotomy, we pool the changes in optimal coupling (relative to the change in control) for the patients and clusters, as seen in Figure 8E and 8F. For both patients and clusters, the distributions show a bias toward lower coupling strengths as induced by the functional connectotomy. The Wilcoxon rank-sum test was computed for the pooled patient and cluster distribution with a highly significant result, indicating a trend toward lower coupling strengths per the means of the distributions. Effectively, there is a general trend indicating that tumors contribute to higher coupling strengths, though, as expected, there is considerable variability between patients.

## DISCUSSION

We found that the optimal parameter fit of the Hopf whole-brain model to phase-based functional connectivity is invariant when $K∼\sqrt{\lambda }$ for *λ* ≥ 0. That is, the phase correlations of the simulated signal remain unchanged as the coupling strength scales with the square root of the excitability (Hopf) parameter. This is intuitive, as for isolated Hopf oscillators, the radius of the stable limit cycle arising at the Hopf bifurcation (occurring at *λ* = 0) is given by $|z|=\sqrt{\lambda }$. Mathematically speaking, $K∼\sqrt{\lambda }$ tells us that the phase dynamics does not change as the ratio between the coupling strength (interregional dynamics) and the radius of the self-sustained, local oscillations (intraregional dynamics) is constant. Moreover, we find the optimal fit to functional connectivity at positive excitability parameters.

Contrary to common modeling assumptions (Freyer et al., 2011; Freyer, Roberts, Ritter, & Breakspear, 2012; Robinson, Rennie, & Rowe, 2002), we did not find that the optimal parameter fit of the whole-brain model was close to criticality, in the sense that the local dynamics are *not* close to the Hopf bifurcation point (*λ* = 0). Indeed, we found the optimal fit to be arbitrary as long as the model was parameterized beyond criticality, with $K∼\sqrt{\lambda }$ for λ ≥ 0. To be more precise, in our optimal fit, the individual local dynamics sustain oscillations independently of network interactions (the network interactions still affect the oscillations, but are not necessary to produce oscillatory signals locally). In most modeling studies, the parameters are set so that the individual oscillators can only sustain oscillations with the aid of network interactions. That is, the parameters are set close to a critical (bifurcation) point, which in our case would be λ ≤ 0. This assumption (λ ≤ 0) is based on the hypothesis that the brain is a dynamical system operating at criticality (Cocchi, Gollo, Zalesky, & Breakspear, 2017). However, in our case, we have shown that the static, time-averaged functional connectivity—as measured by phase correlations—is better captured beyond criticality as defined by the Hopf bifurcation. Nonetheless, when considering the changes in phase correlation as a function of coupling strength, there is another sense of criticality as viewed through the lens of a phase transition (Cabral, Kringelbach, & Deco, 2014). For example, for low coupling strengths, there will be no correlation patterns. However, patterns will arise as the coupling strength is increased, with an optimal coupling strength at intermediary values. In recent years, dynamic functional connectivity has found a growing interest, which reveals the temporal evolution of functional connectivity. It seems likely that parameterizing the bifurcation parameter far beyond criticality *λ* ≫ 0 will *not* reproduce the switching between correlation patterns as observed experimentally with dynamic functional connectivity metrics (Hansen, Battaglia, Spiegler, Deco, & Jirsa, 2015; Kringelbach & Deco, 2020; Vohryzek, Cabral, Vuust, Deco, & Kringelbach, 2022). Although noise has been shown to be key to reproducing functional connectivity as measured by fMRI (Deco, Jirsa, McIntosh, Sporns, & Kötter, 2009), it is not clear whether noise improves fit for E/MEG-measured neural oscillations, which operate at much faster frequencies. However, future studies of the whole-brain dynamics of glioma should incorporate noise if these models are to be fit to fMRI or dynamical functional connectivity in general. Delays in neural communication can be incorporated in whole-brain models and may introduce, together with noise, metastable dynamical phenomena (Cabral et al., 2022; Deco et al., 2009). As such, future glioma modeling studies may introduce delays to understand pathological changes in metastable behavior beyond static resting-state functional connectivity. Another crucial point is the choice of coupling function. In this study, a sigmoidal coupling (Alexandersen et al., 2023; Goriely et al., 2020) was used as opposed to diffusive coupling (Deco, Kringelbach, Jirsa, & Ritter, 2017). Moreover, recent work shows that different frequency bands may be produced by metastable nodes in a noisy whole-brain model for high coupling strength regimes (Cabral et al., 2022; Castaldo et al., 2023). Whether a similar $K∼\sqrt{\lambda }$ scaling exists for diffusive coupling or for dynamical regimes with multistable modes is not known.

Moreover, as revealed by scaling the Hopf whole-brain uniformly by $\sqrt{\lambda }$, the excitability (Hopf) parameter acts as the time scale of the amplitude evolution for positive *λ*. As such, when *λ* is very high, the amplitude dynamics quickly adapt to the network interactions, whereas when *λ* is low, the amplitude dynamics is slow to adapt. As such, when *λ* is high, there is less fluctuation in the simulated signal amplitudes; the amplitude has little effect on the phase dynamics. Whereas, for small *λ*, the amplitude experiences more fluctuations; the amplitude affects the phase dynamics. As such, we suspect the Hopf whole-brain model to behave similarly to a network of Kuramoto oscillators for high *λ* and that the square root scaling $K∼\sqrt{\lambda }$ does not hold for small *λ* (the latter can be seen in Figure 3). It is, therefore, reasonable to assume that amplitude-based metrics—as opposed to phase-based metrics such as PLI—of functional connectivity will not be invariant under $K∼\sqrt{\lambda }$, as λ determines the timescale of amplitude dynamics. Nonetheless, it is still unclear whether a positive or negative λ will reproduce more realistic amplitude correlations.

The Hopf whole-brain model is a phenomenological model. However, it has the advantage of using the Hopf normal form to describe the local (regional) dynamics. As such, close to criticality (Hopf bifurcation point at *λ* = 0), the Hopf normal describes the generic nonlinear behavior close to any Hopf bifurcation. Therefore, we can expect other whole-brain models employing more complicated biophysical neural mass models to behave similarly whenever they are parameterized close to a Hopf bifurcation point. However, we found the optimal fit of the Hopf whole-brain model to be arbitrarily beyond the Hopf bifurcation point. As such, it is not clear whether similar scaling laws between interregional (global) and intraregional (local) dynamics will exist in more complicated neural masses. It would be interesting to see whether a scaling law exists, for example, for the Wilson-Cowan model by performing a similar grid search for the coupling strength and a parameter known to traverse a Hopf bifurcation. In contrast to our study, more biophysical neural mass models have been used to provide insights into the whole-brain dynamics of glioma patients (Aerts et al., 2016, 2018, 2020; van Dellen et al., 2013).

Contrary to our results, studies by Aerts et al. (2018, 2020) do not find significantly different optimal coupling strengths between glioma patients and control subjects. However, the coupling parameter used in Aerts et al. (2018) is more similar to the structural connectivity scaling parameter *C*, for which we do not expect any difference between cohorts, as we see that the structural scaling is underdetermined with respect to functional connectivity (see Supporting Information Figure S1). In these studies, a more complex neural mass model—the reduced Wong-Wang model—was used to fit fMRI functional connectivity. In addition to structural connectivity scaling, local inhibitory parameters were also tuned to guarantee biologically realistic firing rates. The local inhibitory strength was decreased for tumor regions and increased for nontumor regions. The exact analogy for the excitability parameter of the Hopf oscillator is not immediately clear in the reduced Wang-Wong model. However, if tuning the local inhibitory connections decreases the contribution of local dynamics to the emergent whole-brain dynamics, then we would expect the normalized coupling strength to increase as well. Similarly to the study by Aerts et al. (2020), we find that the whole-brain model is quite robust to changes in dynamics after functional connectotomy, though we find a general trend toward lower coupling strengths. That is, the relative contribution of the tumor regions to the distribution of optimal parameters is small, though existent. As the tumor-related empirical data are removed in the functional connectotomy, we observe generally lower coupling strengths. Due to the large variation between patients, it is difficult to make precise assertions about the role of tumors in functional connectivity changes, as larger cohort sizes are needed. Moreover, it should be noted that the changes in global parameters found in this study may arise in a number of ways. Whether the induced changes in neuronal activity are truly global or local is hard to address when fitting global parameters only. However, functional connectotomy suggests that tumor regions contribute to the observed changes in global brain dynamics. Subsequent research will benefit from further investigating the contributions of nontumor regions to global neuronal dynamics. As such, future work should focus on the fitting of parameters local to each brain region to further understand the changes in neuronal activity induced by glioma. The optimal Hopf whole-brain model fit does not show the overall decrease in functional connectivity and changes in clustering and centrality as observed empirically in glioma patients (see Supporting Information Figure S2). Future modeling studies on the whole-brain dynamics need to be precautioned of correlated parameters and account for variation in optimal parameters over initial conditions when applying these models to investigate changes in brain dynamics before and after surgery (Lang et al., 2023). Furthermore, the personalization of the Hopf whole-brain model presented here is limited. The structural connectome and regional intrinsic frequencies were all determined using averages from the healthy cohort (obtaining and analyzing diffusion MRI from glioma patients is particularly difficult due to disease burden and tumor-related scan artifacts). As such, the current study focuses on finding model parameter differences induced by the deviations in functional connectivity from the control average. Although it is known that subject-specific structural connectivity does not improve fit (Aerts et al., 2018; Jirsa et al., 2017), it is unclear whether personalizing the intrinsic frequencies will. Further personalization of whole-brain models may aid in identifying deviations in brain dynamics more accurately in glioma patients.

In conclusion, we showed that the phase dynamics of the Hopf whole-brain model is largely determined by the ratio of interregional and intraregional dynamics, where the ratio is shifted toward interregional dynamics in glioma patients. More precisely, the phase dynamics is unchanged as the coupling strength grows with the square root of the excitability (Hopf) parameter. At the individual and population-based level, we find that glioma patients exhibit whole-brain dynamics with stronger interregional coupling than controls. Moreover, the tumor regions of individual patients contribute to this increase even though there is considerable variation across patients. In summary, we demonstrate an intimate link between global and local dynamics in whole-brain modeling and show that the contribution of global and local dynamics to emergent whole-brain dynamics is disrupted by the presence of tumors in glioma patients.

## ACKNOWLEDGMENTS

We thank Lucas C. Breedt, Chris Vriend, and Marike van Lingen for their help with data acquisition and preprocessing.

## SUPPORTING INFORMATION

Supporting information for this article is available at https://doi.org/10.1162/netn_a_00426.

## AUTHOR CONTRIBUTIONS

Christoffer G. Alexandersen: Data curation; Formal analysis; Investigation; Methodology; Software; Validation; Visualization; Writing – original draft; Writing – review & editing. Linda Douw: Conceptualization; Data curation; Formal analysis; Investigation; Methodology; Writing – original draft; Writing – review & editing. Mona L. M. Zimmermann: Data curation; Formal analysis; Investigation. Christian Bick: Conceptualization; Formal analysis; Investigation; Methodology; Supervision; Writing – original draft; Writing – review & editing. Alain Goriely: Conceptualization; Formal analysis; Investigation; Methodology; Supervision; Writing – original draft; Writing – review & editing.

## FUNDING INFORMATION

Alain Goriely, Engineering and Physical Sciences Research Council (https://dx.doi.org/10.13039/501100000266), Award ID: EP/R020205/1. Linda Douw, Nederlandse Organisatie voor Wetenschappelijk Onderzoek (https://dx.doi.org/10.13039/501100003246), Award ID: NWO Vidi 198.015. Mona Zimmermann, KWF Kankerbestrijding (https://dx.doi.org/10.13039/501100004622), Award ID: KWF project 12885.

## Supplementary Material



## TECHNICAL TERMS

Magnetoencephalography (MEG): A noninvasive imaging method measuring changes in magnetic field due to the electric activity of neurons.
Functional connectivity: Correlations in neural activity across pairs of brain regions, often represented in a network.
Glioma: A brain tumor of cancerous glial cells.
Whole-brain model: A computational model of neuronal activity on the scale of the whole brain.
Craniotomy: Medical procedure opening the skull giving direct access to the brain. Used for surgically removing brain regions.
Structural connectivity: A network of anatomical connections typically derived from diffusion MRI.
Hopf oscillator: A minimal mathematical model for oscillatory phenomena.
Phase-lag index: A metric of functional connectivity used to measure synchronization between brain regions.
Scaling law: A quantitative relationship between two parameters that is essential for observing a given feature.
Interregional coupling: A whole-brain model parameter determining how strongly brain regions influence each other.
Intraregional excitability: A whole-brain model parameter determining how excitable brain regions are to input.

## References

[bib1] Aerts, H., Fias, W., Caeyenberghs, K., & Marinazzo, D. (2016). Brain networks under attack: Robustness properties and the impact of lesions. Brain, 139(12), 3063–3083. 10.1093/brain/aww194, 27497487

[bib2] Aerts, H., Schirner, M., Dhollander, T., Jeurissen, B., Achten, E., Van Roost, D., … Marinazzo, D. (2020). Modeling brain dynamics after tumor resection using the virtual brain. NeuroImage, 213, 116738. 10.1016/j.neuroimage.2020.116738, 32194282

[bib3] Aerts, H., Schirner, M., Jeurissen, B., Van Roost, D., Achten, E., Ritter, P., & Marinazzo, D. (2018). Modeling brain dynamics in brain tumor patients using the virtual brain. eNeuro, 5(3), ENEURO.0083-18.2018. 10.1523/ENEURO.0083-18.2018, 29911173 PMC6001263

[bib4] Alexandersen, C. G., de Haan, W., Bick, C., & Goriely, A. (2023). A multi-scale model explains oscillatory slowing and neuronal hyperactivity in Alzheimer’s disease. Journal of The Royal Society Interface, 20(198), 20220607. 10.1098/rsif.2022.0607, 36596460 PMC9810432

[bib5] Alstott, J., Breakspear, M., Hagmann, P., Cammoun, L., & Sporns, O. (2009). Modeling the impact of lesions in the human brain. PLoS Computational Biology, 5(6), e1000408. 10.1371/journal.pcbi.1000408, 19521503 PMC2688028

[bib6] Andersson, J. L. R., Skare, S., & Ashburner, J. (2003). How to correct susceptibility distortions in spin-echo echo-planar images: Application to diffusion tensor imaging. NeuroImage, 20(2), 870–888. 10.1016/S1053-8119(03)00336-7, 14568458

[bib7] Anticevic, A., Gancsos, M., Murray, J. D., Repovs, G., Driesen, N. R., Ennis, D. J., … Corlett, P. R. (2012). NMDA receptor function in large-scale anticorrelated neural systems with implications for cognition and schizophrenia. Proceedings of the National Academy of Sciences, 109(41), 16720–16725. 10.1073/pnas.1208494109, 23012427 PMC3478611

[bib8] Bartolomei, F., Bosma, I., Klein, M., Baayen, J. C., Reijneveld, J. C., Postma, T. J., … Stam, C. J. (2006a). Disturbed functional connectivity in brain tumour patients: Evaluation by graph analysis of synchronization matrices. Clinical Neurophysiology, 117(9), 2039–2049. 10.1016/j.clinph.2006.05.018, 16859985

[bib9] Bartolomei, F., Bosma, I., Klein, M., Baayen, J. C., Reijneveld, J. C., Postma, T. J., … Stam, C. J. (2006b). How do brain tumors alter functional connectivity? A magnetoencephalography study. Annals of Neurology, 59(1), 128–138. 10.1002/ana.20710, 16278872

[bib10] Bosma, I., Douw, L., Bartolomei, F., Heimans, J. J., van Dijk, B. W., Postma, T. J., … Klein, M. (2008). Synchronized brain activity and neurocognitive function in patients with low-grade glioma: A magnetoencephalography study. Neuro-Oncology, 10(5), 734–744. 10.1215/15228517-2008-034, 18650489 PMC2666250

[bib11] Bosma, I., Reijneveld, J. C., Klein, M., Douw, L., van Dijk, B. W., Heimans, J. J., & Stam, C. J. (2009). Disturbed functional brain networks and neurocognitive function in low-grade glioma patients: A graph theoretical analysis of resting-state MEG. Nonlinear Biomedical Physics, 3(1), 9. 10.1186/1753-4631-3-9, 19698149 PMC2745411

[bib12] Breedt, L. C., Santos, F. A. N., Hillebrand, A., Reneman, L., van Rootselaar, A.-F., Schoonheim, M. M., … Douw, L. (2023). Multimodal multilayer network centrality relates to executive functioning. Network Neuroscience, 7(1), 299–321. 10.1162/netn_a_00284, 37339322 PMC10275212

[bib13] Cabral, J., Castaldo, F., Vohryzek, J., Litvak, V., Bick, C., Lambiotte, R., … Deco, G. (2022). Metastable oscillatory modes emerge from synchronization in the brain spacetime connectome. Communications Physics, 5, 184. 10.1038/s42005-022-00950-y, 38288392 PMC7615562

[bib14] Cabral, J., Fernandes, H. M., Van Hartevelt, T. J., James, A. C., Kringelbach, M. L., & Deco, G. (2013). Structural connectivity in schizophrenia and its impact on the dynamics of spontaneous functional networks. Chaos, 23(4), 046111. 10.1063/1.4851117 24387590

[bib15] Cabral, J., Hugues, E., Sporns, O., & Deco, G. (2011). Role of local network oscillations in resting-state functional connectivity. NeuroImage, 57(1), 130–139. 10.1016/j.neuroimage.2011.04.010, 21511044

[bib16] Cabral, J., Kringelbach, M. L., & Deco, G. (2014). Exploring the network dynamics underlying brain activity during rest. Progress in Neurobiology, 114, 102–131. 10.1016/j.pneurobio.2013.12.005, 24389385

[bib17] Castaldo, F., Páscoa Dos Santos, F., Timms, R. C., Cabral, J., Vohryzek, J., Deco, G., … Litvak, V. (2023). Multi-modal and multi-model interrogation of large-scale functional brain networks. NeuroImage, 277, 120236. 10.1016/j.neuroimage.2023.120236, 37355200 PMC10958139

[bib18] Cocchi, L., Gollo, L. L., Zalesky, A., & Breakspear, M. (2017). Criticality in the brain: A synthesis of neurobiology, models and cognition. Progress in Neurobiology, 158, 132–152. 10.1016/j.pneurobio.2017.07.002, 28734836

[bib19] Deco, G., Jirsa, V., McIntosh, A. R., Sporns, O., & Kötter, R. (2009). Key role of coupling, delay, and noise in resting brain fluctuations. Proceedings of the National Academy of Sciences, 106(25), 10302–10307. 10.1073/pnas.0901831106, 19497858 PMC2690605

[bib20] Deco, G., Jirsa, V. K., & McIntosh, A. R. (2011). Emerging concepts for the dynamical organization of resting-state activity in the brain. Nature Reviews Neuroscience, 12(1), 43–56. 10.1038/nrn2961, 21170073

[bib21] Deco, G., & Kringelbach, M. L. (2014). Great expectations: Using whole-brain computational connectomics for understanding neuropsychiatric disorders. Neuron, 84(5), 892–905. 10.1016/j.neuron.2014.08.034, 25475184

[bib22] Deco, G., Kringelbach, M. L., Jirsa, V. K., & Ritter, P. (2017). The dynamics of resting fluctuations in the brain: Metastability and its dynamical cortical core. Scientific Reports, 7(1), 3095. 10.1038/s41598-017-03073-5, 28596608 PMC5465179

[bib23] Demirtaş, M., Falcon, C., Tucholka, A., Gispert, J. D., Molinuevo, J. L., & Deco, G. (2017). A whole-brain computational modeling approach to explain the alterations in resting-state functional connectivity during progression of Alzheimer’s disease. NeuroImage: Clinical, 16, 343–354. 10.1016/j.nicl.2017.08.006, 28861336 PMC5568172

[bib24] Derks, J., Kulik, S. D., Numan, T., de Witt Hamer, P. C., Noske, D. P., Klein, M., … Douw, L. (2021). Understanding global brain network alterations in glioma patients. Brain Connectivity, 11(10), 865–874. 10.1089/brain.2020.0801, 33947274

[bib25] Derks, J., Reijneveld, J. C., & Douw, L. (2014). Neural network alterations underlie cognitive deficits in brain tumor patients. Current Opinion in Oncology, 26(6), 627–633. 10.1097/CCO.0000000000000126, 25188475

[bib26] Esposito, R., Mattei, P. A., Briganti, C., Romani, G. L., Tartaro, A., & Caulo, M. (2012). Modifications of default-mode network connectivity in patients with cerebral glioma. PLOS ONE, 7(7), e40231. 10.1371/journal.pone.0040231, 22808124 PMC3392269

[bib27] Fox, M. E., & King, T. Z. (2018). Functional connectivity in adult brain tumor patients: A systematic review. Brain Connectivity, 8(7), 381–397. 10.1089/brain.2018.0623, 30141339

[bib28] Freyer, F., Roberts, J. A., Becker, R., Robinson, P. A., Ritter, P., & Breakspear, M. (2011). Biophysical mechanisms of multistability in resting-state cortical rhythms. Journal of Neuroscience, 31(17), 6353–6361. 10.1523/JNEUROSCI.6693-10.2011, 21525275 PMC6622680

[bib29] Freyer, F., Roberts, J. A., Ritter, P., & Breakspear, M. (2012). A canonical model of multistability and scale-invariance in biological systems. PLoS Computational Biology, 8(8), e1002634. 10.1371/journal.pcbi.1002634, 22912567 PMC3415415

[bib30] Ghumman, S., Fortin, D., Noel-Lamy, M., Cunnane, S. C., & Whittingstall, K. (2016). Exploratory study of the effect of brain tumors on the default mode network. Journal of Neuro-Oncology, 128(3), 437–444. 10.1007/s11060-016-2129-6, 27090892

[bib31] Goriely, A., Kuhl, E., & Bick, C. (2020). Neuronal oscillations on evolving networks: Dynamics, damage, degradation, decline, dementia, and death. Physical Review Letters, 125(12), 128102. 10.1103/PhysRevLett.125.128102, 33016724

[bib32] Guggisberg, A. G., Honma, S. M., Findlay, A. M., Dalal, S. S., Kirsch, H. E., Berger, M. S., & Nagarajan, S. S. (2008). Mapping functional connectivity in patients with brain lesions. Annals of Neurology, 63(2), 193–203. 10.1002/ana.21224, 17894381 PMC3646715

[bib33] Hansen, E. C. A., Battaglia, D., Spiegler, A., Deco, G., & Jirsa, V. K. (2015). Functional connectivity dynamics: Modeling the switching behavior of the resting state. NeuroImage, 105, 525–535. 10.1016/j.neuroimage.2014.11.001, 25462790

[bib34] Harris, R. J., Bookheimer, S. Y., Cloughesy, T. F., Kim, H. J., Pope, W. B., Lai, A., … Ellingson, B. M. (2014). Altered functional connectivity of the default mode network in diffuse gliomas measured with pseudo-resting state fMRI. Journal of Neuro-Oncology, 116(2), 373–379. 10.1007/s11060-013-1304-2, 24234804 PMC6763342

[bib35] Hillebrand, A., Barnes, G. R., Bosboom, J. L., Berendse, H. W., & Stam, C. J. (2012). Frequency-dependent functional connectivity within resting-state networks: An atlas-based MEG beamformer solution. NeuroImage, 59(4), 3909–3921. 10.1016/j.neuroimage.2011.11.005 22122866 PMC3382730

[bib36] Hillebrand, A., Tewarie, P., van Dellen, E., Yu, M., Carbo, E. W. S., Douw, L., … Stam, C. J. (2016). Direction of information flow in large-scale resting-state networks is frequency-dependent. Proceedings of the National Academy of Sciences, 113(14), 3867–3872. 10.1073/pnas.1515657113, 27001844 PMC4833227

[bib37] Jalilianhasanpour, R., Beheshtian, E., Sherbaf, G., Sahraian, S., & Sair, H. I. (2019). Functional connectivity in neurodegenerative disorders: Alzheimer’s disease and frontotemporal dementia. Topics in Magnetic Resonance Imaging, 28(6), 317–324. 10.1097/RMR.0000000000000223, 31794504

[bib38] Jeurissen, B., Tournier, J.-D., Dhollander, T., Connelly, A., & Sijbers, J. (2014). Multi-tissue constrained spherical deconvolution for improved analysis of multi-shell diffusion MRI data. NeuroImage, 103, 411–426. 10.1016/j.neuroimage.2014.07.061, 25109526

[bib39] Jirsa, V. K., Proix, T., Perdikis, D., Woodman, M. M., Wang, H., Gonzalez-Martinez, J., … Bartolomei, F. (2017). The virtual epileptic patient: Individualized whole-brain models of epilepsy spread. NeuroImage, 145, 377–388. 10.1016/j.neuroimage.2016.04.049, 27477535

[bib40] Kringelbach, M. L., & Deco, G. (2020). Brain states and transitions: Insights from computational neuroscience. Cell Reports, 32(10), 108128. 10.1016/j.celrep.2020.108128, 32905760

[bib41] Lang, S., Momi, D., Vetkas, A., Santyr, B., Yang, A. Z., Kalia, S. K., … Lozano, A. (2023). Computational modeling of whole-brain dynamics: A review of neurosurgical applications. Journal of Neurosurgery, 140(1), 218–230. 10.3171/2023.5.JNS23250, 37382356

[bib42] Li, S., Hu, N., Zhang, W., Tao, B., Dai, J., Gong, Y., … Lui, S. (2019). Dysconnectivity of multiple brain networks in schizophrenia: A meta-analysis of resting-state functional connectivity. Frontiers in Psychiatry, 10, 482. 10.3389/fpsyt.2019.00482, 31354545 PMC6639431

[bib43] Li, X.-Y., Chen, X., & Yan, C.-G. (2022). Altered cerebral activities and functional connectivity in depression: A systematic review of fMRI studies. Quantitative Biology, 10(4), 366–380. 10.15302/J-QB-021-0270

[bib44] Maas, D. A., & Douw, L. (2023). Multiscale network neuroscience in neuro-oncology: How tumors, brain networks, and behavior connect across scales. Neuro-Oncology Practice, 10(6), 506–517. 10.1093/nop/npad044 38026586 PMC10666814

[bib45] Maesawa, S., Bagarinao, E., Fujii, M., Futamura, M., Motomura, K., Watanabe, H., … Wakabayashi, T. (2015). Evaluation of resting state networks in patients with gliomas: Connectivity changes in the unaffected side and its relation to cognitive function. PLoS One, 10(2), e0118072. 10.1371/journal.pone.0118072, 25659130 PMC4319851

[bib46] Nissen, I. A., Millán, A. P., Stam, C. J., van Straaten, E. C. W., Douw, L., Pouwels, P. J. W., … Hillebrand, A. (2021). Optimization of epilepsy surgery through virtual resections on individual structural brain networks. Scientific Reports, 11(1), 19025. 10.1038/s41598-021-98046-0, 34561483 PMC8463605

[bib47] Pathak, A., Roy, D., & Banerjee, A. (2022). Whole-brain network models: From physics to bedside. Frontiers in Computational Neuroscience, 16, 866517. 10.3389/fncom.2022.866517, 35694610 PMC9180729

[bib48] Robinson, P. A., Rennie, C. J., & Rowe, D. L. (2002). Dynamics of large-scale brain activity in normal arousal states and epileptic seizures. Physical Review E, 65(4), 041924. 10.1103/PhysRevE.65.041924, 12005890

[bib49] Smith, R. E., Tournier, J.-D., Calamante, F., & Connelly, A. (2012). Anatomically-constrained tractography: Improved diffusion MRI streamlines tractography through effective use of anatomical information. NeuroImage, 62(3), 1924–1938. 10.1016/j.neuroimage.2012.06.005, 22705374

[bib50] Smith, R. E., Tournier, J.-D., Calamante, F., & Connelly, A. (2015). SIFT2: Enabling dense quantitative assessment of brain white matter connectivity using streamlines tractography. NeuroImage, 119, 338–351. 10.1016/j.neuroimage.2015.06.092, 26163802

[bib51] Stam, C. J., Nolte, G., & Daffertshofer, A. (2007). Phase lag index: Assessment of functional connectivity from multi channel EEG and MEG with diminished bias from common sources. Human Brain Mapping, 28(11), 1178–1193. 10.1002/hbm.20346, 17266107 PMC6871367

[bib52] Stefanovski, L., Triebkorn, P., Spiegler, A., Diaz-Cortes, M.-A., Solodkin, A., Jirsa, V., … Alzheimer’s Disease Neuroimaging Initiative. (2019). Linking molecular pathways and large-scale computational modeling to assess candidate disease mechanisms and pharmacodynamics in Alzheimer’s disease. Frontiers in Computational Neuroscience, 13, 54. 10.3389/fncom.2019.00054, 31456676 PMC6700386

[bib53] Taylor, P. N., Kaiser, M., & Dauwels, J. (2014). Structural connectivity based whole brain modelling in epilepsy. Journal of Neuroscience Methods, 236, 51–57. 10.1016/j.jneumeth.2014.08.010, 25149109

[bib54] Tournier, J.-D., Smith, R., Raffelt, D., Tabbara, R., Dhollander, T., Pietsch, M., … Connelly, A. (2019). MRtrix3: A fast, flexible and open software framework for medical image processing and visualisation. NeuroImage, 202, 116137. 10.1016/j.neuroimage.2019.116137, 31473352

[bib55] Tzourio-Mazoyer, N., Landeau, B., Papathanassiou, D., Crivello, F., Etard, O., Delcroix, N., … Joliot, M. (2002). Automated anatomical labeling of activations in SPM using a macroscopic anatomical parcellation of the MNI MRI single-subject brain. NeuroImage, 15(1), 273–289. 10.1006/nimg.2001.0978, 11771995

[bib56] van Dellen, E., Douw, L., Hillebrand, A., Ris-Hilgersom, I. H. M., Schoonheim, M. M., Baayen, J. C., … Reijneveld, J. C. (2012). MEG network differences between low- and high-grade glioma related to epilepsy and cognition. PLoS One, 7(11), e50122. 10.1371/journal.pone.0050122, 23166829 PMC3498183

[bib57] van Dellen, E., Hillebrand, A., Douw, L., Heimans, J. J., Reijneveld, J. C., & Stam, C. J. (2013). Local polymorphic delta activity in cortical lesions causes global decreases in functional connectivity. NeuroImage, 83, 524–532. 10.1016/j.neuroimage.2013.06.009, 23769919

[bib58] Virtanen, P., Gommers, R., Oliphant, T. E., Haberland, M., Reddy, T., Cournapeau, D., … SciPy 1.0 Contributors. (2020). SciPy 1.0: Fundamental algorithms for scientific computing in Python. Nature Methods, 17(3), 261–272. 10.1038/s41592-019-0686-2, 32015543 PMC7056644

[bib59] Vohryzek, J., Cabral, J., Vuust, P., Deco, G., & Kringelbach, M. L. (2022). Understanding brain states across spacetime informed by whole-brain modelling. Philosophical Transactions of the Royal Society A, 380(2227), 20210247. 10.1098/rsta.2021.0247, 35599554 PMC9125224

[bib60] Xu, H., Ding, S., Hu, X., Yang, K., Xiao, C., Zou, Y., … Qian, Z. (2013). Reduced efficiency of functional brain network underlying intellectual decline in patients with low-grade glioma. Neuroscience Letters, 543, 27–31. 10.1016/j.neulet.2013.02.062, 23562503

[bib61] Yang, G. J., Murray, J. D., Repovs, G., Cole, M. W., Savic, A., Glasser, M. F., … Anticevic, A. (2014). Altered global brain signal in schizophrenia. Proceedings of the National Academy of Sciences, 111(20), 7438–7443. 10.1073/pnas.1405289111, 24799682 PMC4034208

