# Supplementary material for: Functional connectotomy of a whole-brain model reveals tumor-induced alterations to neuronal dynamics in glioma patients
Source: Netw Neurosci. 2025 Mar 20;9(1):280–302. doi: 10.1162/netn_a_00426 (PMC11949587; doi:10.1162/netn_a_00426)
Supplement: Supplementary file 1 [file netn-9-1-280-s001.pdf]

Supporting Information for

Functional connectotomy of a whole-brain model reveals tumor-induced alterations to neuronal dynamics in glioma patient

Christoffer G. Alexandersen, Linda Douw, Mona L.M. Zimmermann, Christian Bick, Alain Goriely

Computation of the phase-lag index

In this study, we used the *phase-lag index* (PLI) as a measure of functional connectivity [1]. PLI is a measure of the average asymmetry in pair-wise phase differences of oscillatory MEG signals from different brain regions and ranges from 0 (symmetry) to 1 (asymmetry) for each pair of regions.

The phase-lag index is computed in the same way for each cohort. For each cohort, we have S subjects. For each subject $s \in \{1, \dots, S\}$, we have E_s epochs, each consisting of a $N \times M$ matrix, where N is the number of brain regions and M is the number of time points. We denote each matrix by $\hat{x}_{s,e} \in \mathbb{R}^{N \times M}$ with dimensions N and M, where e denotes the epoch and s denotes the subject.

For processing, we apply a Butterworth filter to bandpass the signal into the alpha frequency band at 8–12 Hz. Thus, the Butterworth filter is a function $F : \mathbb{R}^{N \times M} \to \mathbb{R}^{N \times M}$ that can be applied to each epoch. After bandpassing the data, we find the angle of its Hilbert transform $\Theta : \mathbb{R}^{N \times M} \to \mathbb{R}^{N \times M}$. Next, we compute the phase-lag index connectivity. That is, we apply a function $P : \mathbb{R}^{N \times M} \to \mathbb{R}^{N \times N}$ to the data to construct a N-by-N, symmetric, functional connectivity matrix. This phase-lag index transformation is given explicitly by

$$P(\theta)_{ij} = |\langle \text{sign}(\theta_i - \theta_j) \rangle_t| \quad \text{for } i = 1, \dots, N, j = 1, \dots, N, \tag{1}$$

where $\langle \, \rangle_t$ is the time average. We apply this process to compute the PLI for each matrix $\hat{x}_{s,e}$:

$$P(\hat{x}_{s,e}) = P(\Theta(F(\hat{x}_{s,e})))m \tag{2}$$

and compute the average experimental phase-lag index matrix for the entire cohort as

$$P^{\text{exp}} = \frac{1}{S} \sum_{s=1}^{S} \frac{1}{E_s} \sum_{e=1}^{E_s} P(\hat{x}_{s,e}). \tag{3}$$

The experimental PLI matrices are noisy and are thus thresholded to ease the comparison with the noiseless whole-brain model. Thresholding the PLI matrix at a certain percentage $X\%$ means setting all elements in the lower $X\%$ percent to zero as ordered per magnitude. In some cases, we perform median thresholding instead, where all elements of the PLI matrix lower than the median of said matrix are set to zero.

Computation of the Pearson correlation as a goodness-of-fit for the whole-brain model

We interpret the real part of the Hopf oscillator variables x_i as MEG signal and process it in the same manner as the experimental MEG signal. That is, we bandpass the signal into the alpha frequency band (8-12Hz) using a Butterworth filter (denoted by the function F) and subsequently apply the Hilbert transform to find the angles of the oscillators (denoted by the function Θ). We then compute the pairwise time-averaged phase-lag index of the processed simulated signal and compute the Pearson correlation of the simulated PLI matrix and the experimental PLI matrix.

Let $x \in \mathbb{R}^{N \times M}$ denote the simulated time series of N regions over M time points. Note that x is a function of the parameters and the initial conditions $z(0) = z_0$ of the whole-brain model. As such, the simulated phase-lag index is $P^{\text{sim}} =$

$P(\Theta(F(x(K, \lambda, C, x_0))))$, with P, F, and Θ defined above. Remembering that P^{exp} denotes the experimental MEG PLI matrix averaged over epochs and subjects, the objective function is

$$f(K, \lambda, C, z_0; P^{\text{exp}}) = \frac{\left(\sum_{ij}^{N} P_{ij}^{\text{sim}} - \langle P^{\text{sim}} \rangle\right)\left(\sum_{ij}^{N} P_{ij}^{\text{exp}} - \langle P^{\text{exp}} \rangle\right)}{\sqrt{\sum_{ij}^{N} \left(P_{ij}^{\text{sim}} - \langle P^{\text{sim}} \rangle\right)^2 \left(P_{ij}^{\text{exp}} - \langle P^{\text{exp}} \rangle\right)^2}}, \tag{4}$$

where $\langle\rangle$ denotes the average over all matrix entries. As the objective function depends on the initial conditions z_0, we average the objective function over several runs with random initial conditions. For random initial conditions, each oscillator's initial state z_i for $i = 1, \ldots, N$ is a random point in the unit disk $|z_i| \leq 1$ picked with uniform probability.

Rescaling the Hopf whole-brain model unveils the normalized coupling strength

By scaling the variables of the Hopf whole-brain model by $\tilde{x}_i = \sqrt{\lambda} x_i, \tilde{y}_i = \sqrt{\lambda} y_i$ for $i = 1 \ldots N$, we obtain the following equivalent system

$$\frac{d\tilde{x}_i}{dt} = \lambda \tilde{x}_i - \omega_i \tilde{y}_i - \lambda \tilde{x}_i (\tilde{x}_i^2 + \tilde{y}_i^2) + \frac{K}{\sqrt{\lambda}} \tanh\left(C\sqrt{\lambda} \sum_{j=1}^{N} w_{ij} \tilde{x}_j\right), \tag{5}$$

$$\frac{d\tilde{y}_i}{dt} = \lambda \tilde{y}_i + \omega_i \tilde{x}_i - \lambda \tilde{y}_i (\tilde{x}_i^2 + \tilde{y}_i^2), \tag{6}$$

for $\lambda > 0$. The scaled system above is equivalent to a system coupling Hopf oscillators with a stable limit cycle of radius 1 with a modified coupling constant $K/\sqrt{\lambda}$. Additionally, the excitability parameter λ does not scale the radius of the limit cycle as in the original system. Instead, it is the timescale of the amplitude dynamics.

Structural connectivity scaling

For varying levels of excitability values, we find that the goodness of fit of the Hopf whole-brain model is largely insensitive to the scaling of the structural connectome. As demonstrated in Figure S1, the scaling of the structural connectome just needs to be of sufficient magnitude to achieve optimal fit.

Network metrics of simulated functional connectivity

The network metrics of the simulated phase-lag index connectivity are not similar to the metrics found for empirical phase-lag index connectivity for optimal model parameters. There are only minor differences in network metrics between the optimal fit for the glioma and the control cohort, as shown in Fig. S2.

The effect of thresholding the empirical functional connectivity

The difference in optimal normalized coupling strength between the glioma and control cohort is, for the most part, positive across varying degrees of thresholding (see Figure S4 and S5). Both cohorts' functional connectivity fit has its peak around 97% thresholding, which is the threshold used for the simulations in the Results unless specified otherwise.

When thresholding the glioma and control cohort by their median values (not the same thresholding value between the cohorts), we obtain similar results as for the optimal thresholding value (as shown in the Results), though with smaller differences in optimal coupling strength between the cohorts (see Figure S6).

Pearson correlations of control and patients

The Pearson correlations of the control and patients over initial conditions are shown below in Fig.S7. We compute the Pearson correlation between the structural connectome to the empirical functional connectomes as a benchmark for the simulated functional connectivity. We see that the patient-specific and average control Pearson correlation are consequently higher than the Pearson correlation to the average control structural connectome, meaning that the whole-brain models are better at capturing functional connectivity than the structural connectome. Furthermore, the Pearson correlation either remains

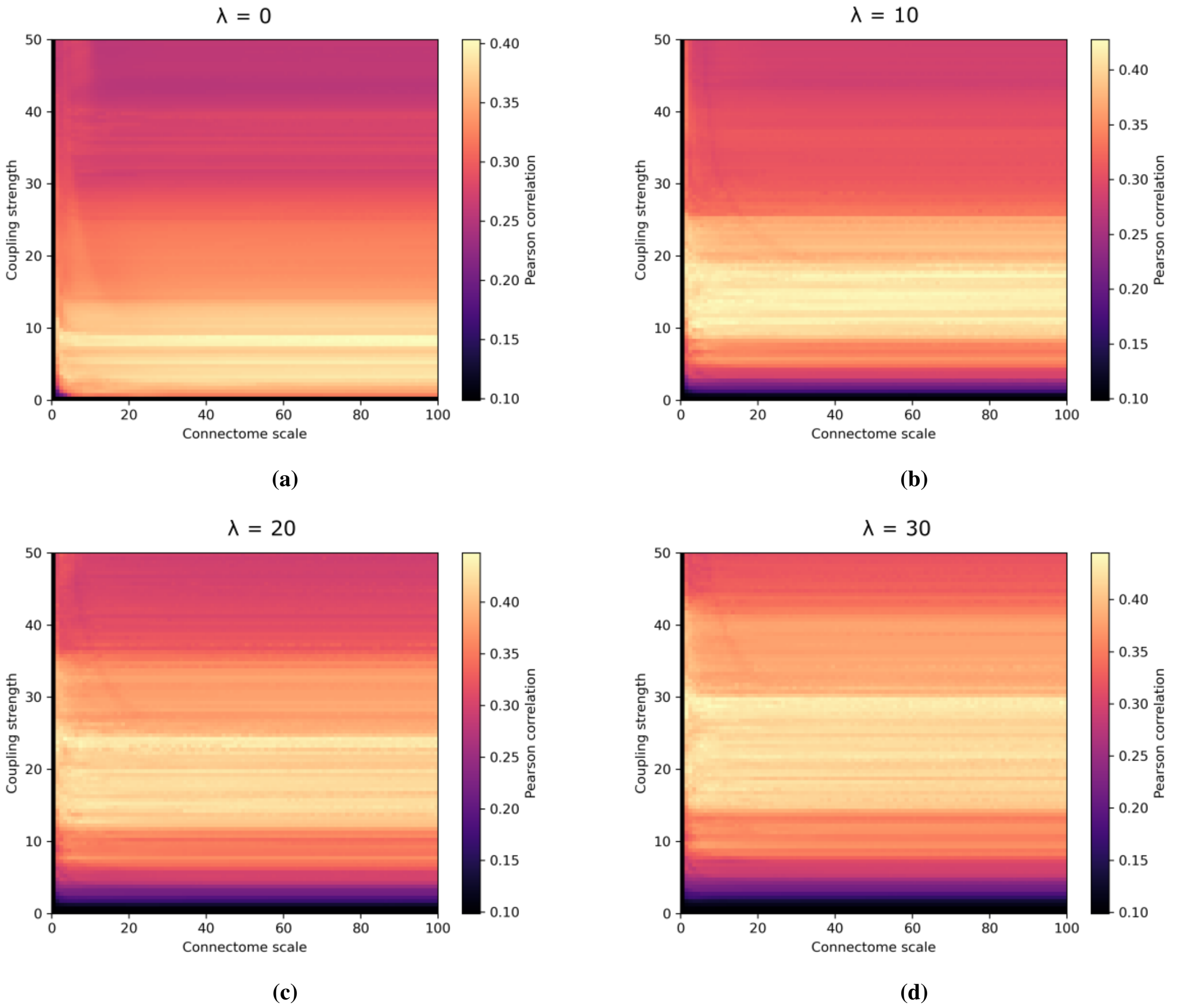

Figure S1: Grid search over coupling strength and connectome scaling for different excitability values for a set, random initial condition of the Hopf whole-brain model. Each pixel corresponds to the Pearson correlation between simulated and experimental (healthy control group) phase-lag index connectivity derived from MEG. As shown, the connectome scaling does not alter the model fit when sufficiently large, when varying the coupling strength and global excitability parameter λ. (a) $\lambda = 0$, (b) $\lambda = 10$, (c) $\lambda = 20$, (d) $\lambda = 30$

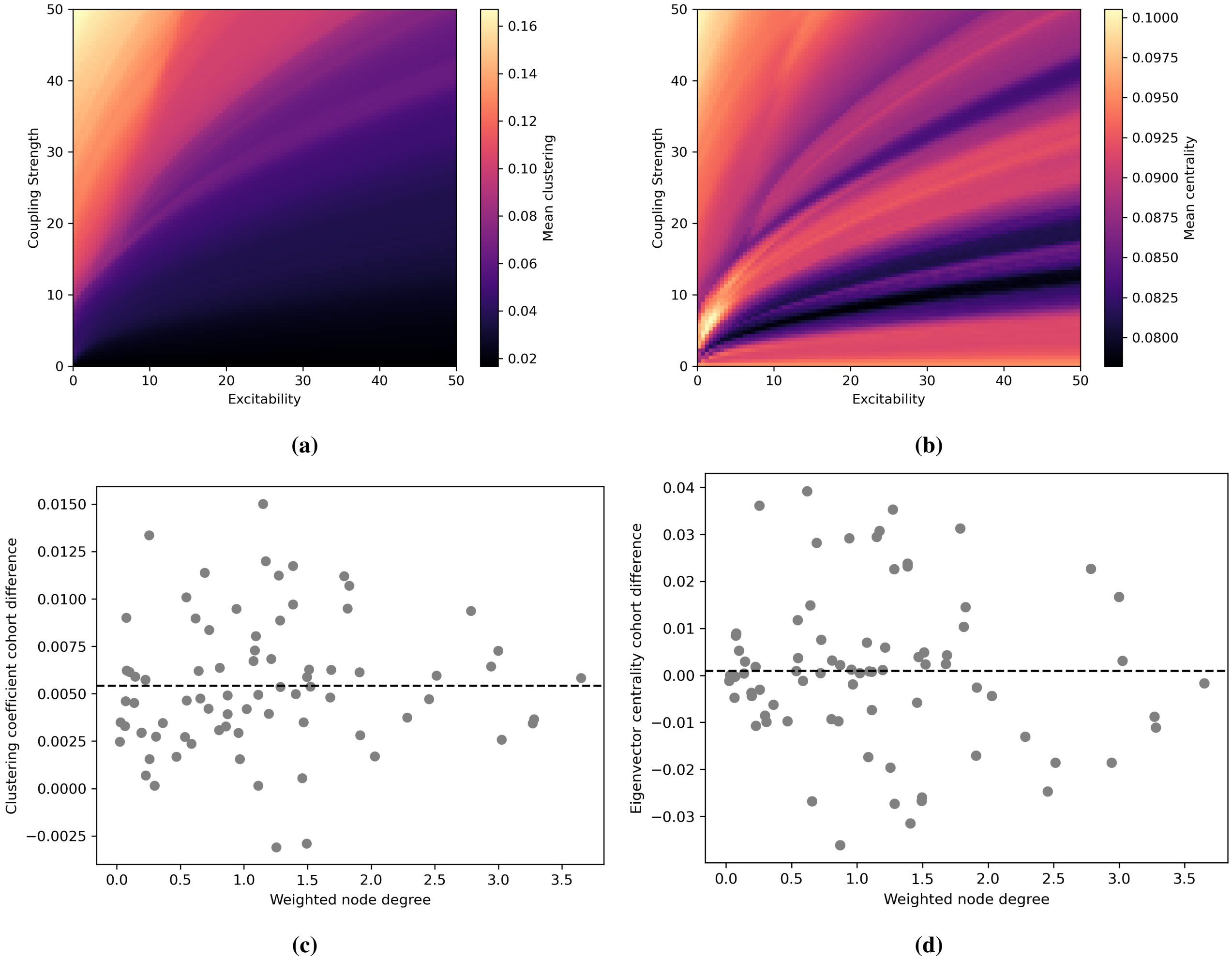

Figure S2: Network metrics of the simulated phase-lag index connectivity. (a) The average clustering coefficient of synthetic functional connectivity (phase-lag index) of the Hopf whole-brain model over the coupling strength and excitability parameter. The experimental clustering coefficient is around 0.2 for the healthy cohort. (b) The average eigenvector centrality over coupling strength and excitability. The empirical average eigenvector centrality is close to 0.05. (c,d) The difference in the simulated clustering coefficient and eigenvector centrality between the optimal model fit for the glioma and control group per node (plotted by their weighted structural node degree). The black stippled line shows the average difference, both of which are close to zero.

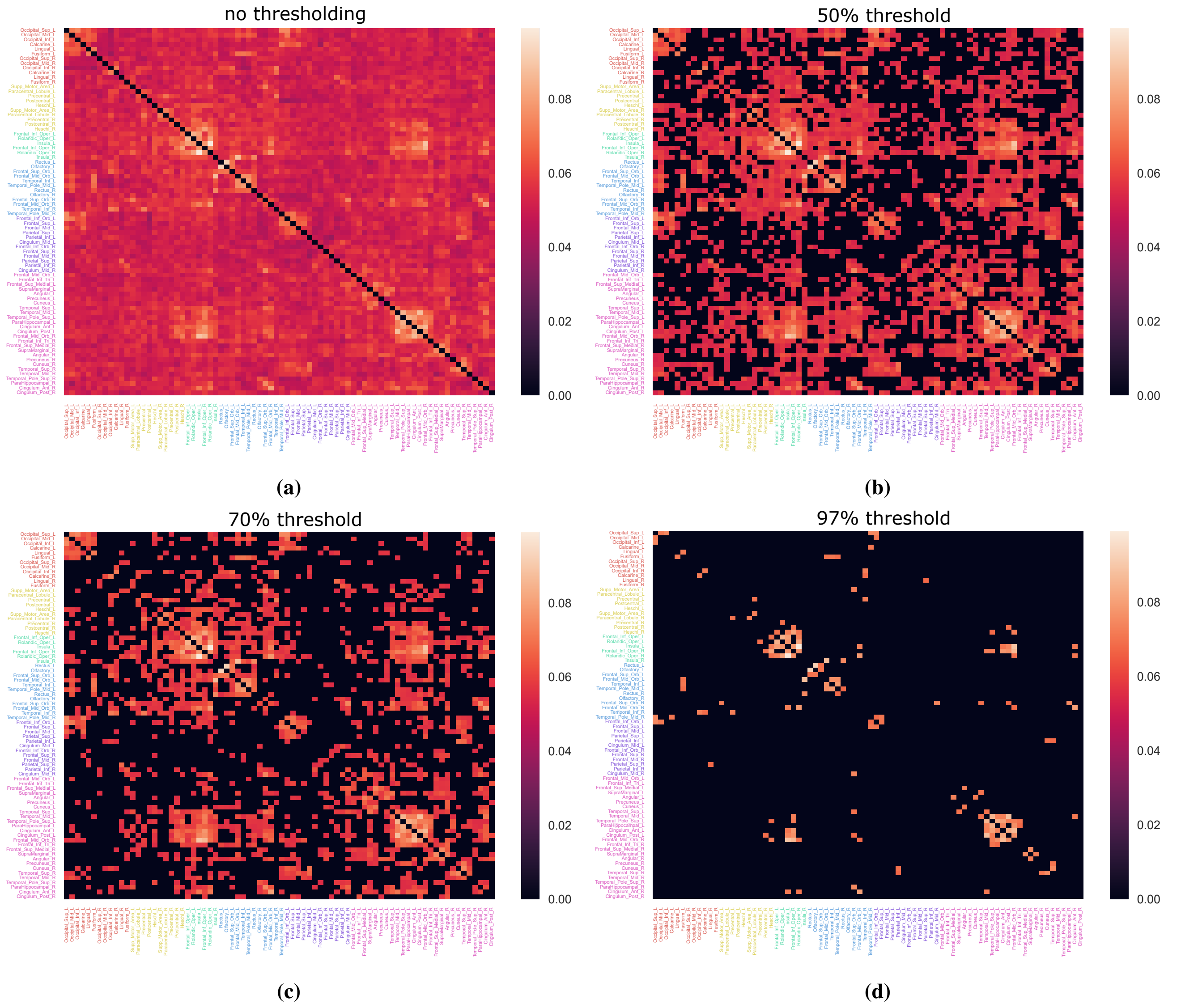

Figure S3: The post-processed average experimental phase-lag index connectome of the control group for varying levels of thresholding. During thresholding, all elements that belong to the lower X% are set to zero, while others are kept at their original value. *(a)* before thresholding, *(b)* 50% (median threshold), *(c)* 70%, *(d)* 97% (the percentage giving the highest goodness of fit to simulated data).

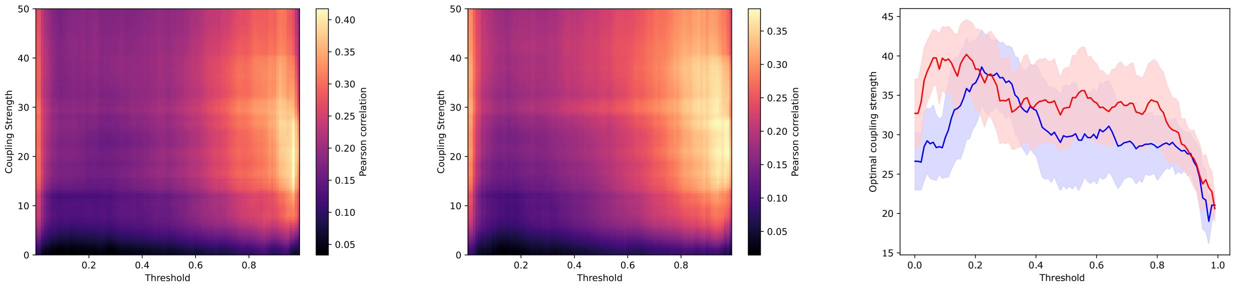

Figure S4: Grid searches showing the effect of coupling strength and thresholding of experimental phase-lag connectomes on the Pearson correlation between simulated and experimental phase-lag connectivity. The grid search matrices show the average of 300 initial conditions for the Hopf whole-brain model. *(a)* Grid search showing the fit to the average healthy phase-lag connectivity. *(b)* Grid search showing the fit to the average glioma patient phase-lag connectivity. *(c)* Plot showing the average (solid line) and standard deviation (shaded region) of the optimal coupling strength per experimental threshold for the control (blue) and glioma (red) cohort over initial conditions.

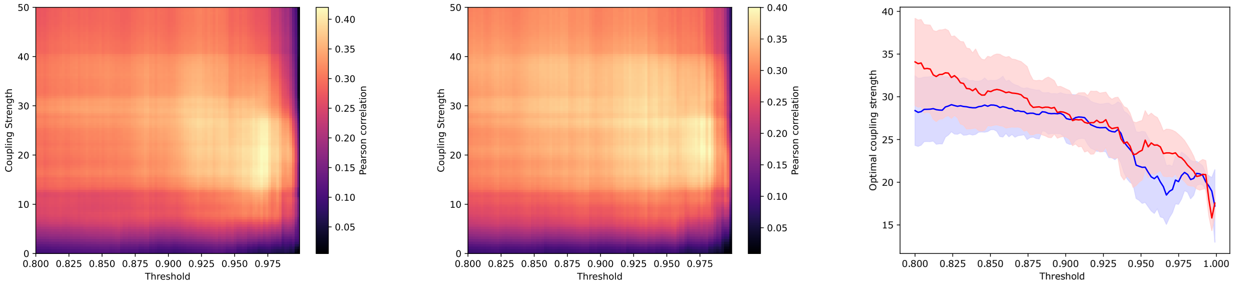

Figure S5: This plot is identical to Fig. S4 but zoomed in for higher threshold values.

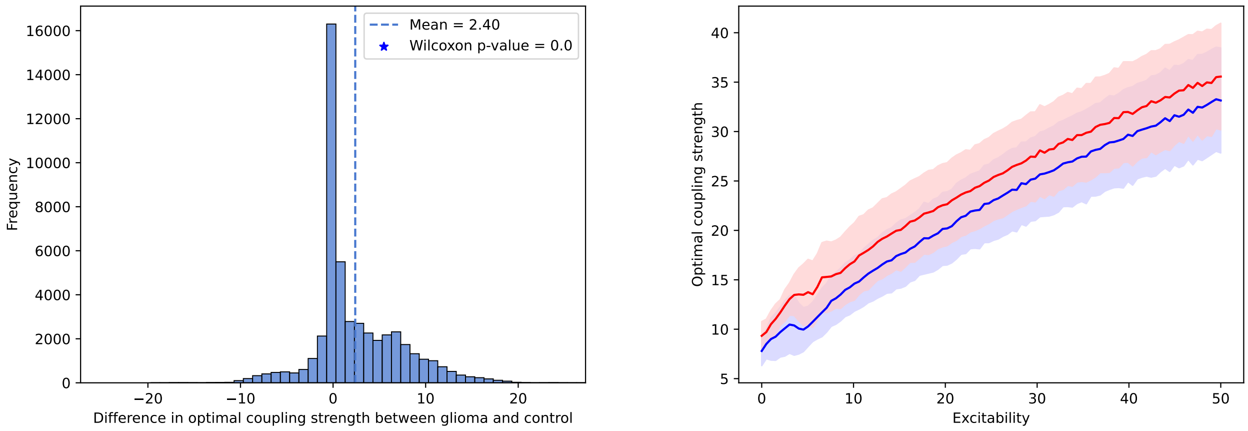

(a) **(b)**

Figure S6: Optimal parameters for the Hopf whole-brain model fitted to PLI functional connectivity data when thresholded by their median value. The model was fitted to average PLI over each group for 100 randomized initial conditions. (a) The difference in optimal coupling strength (pooled over all excitability parameter values for 100 different initial conditions. (b) The mean (solid line) and standard deviation (shaded region) of optimal coupling strength per excitability parameter for the healthy (blue) and glioma cohort (red).

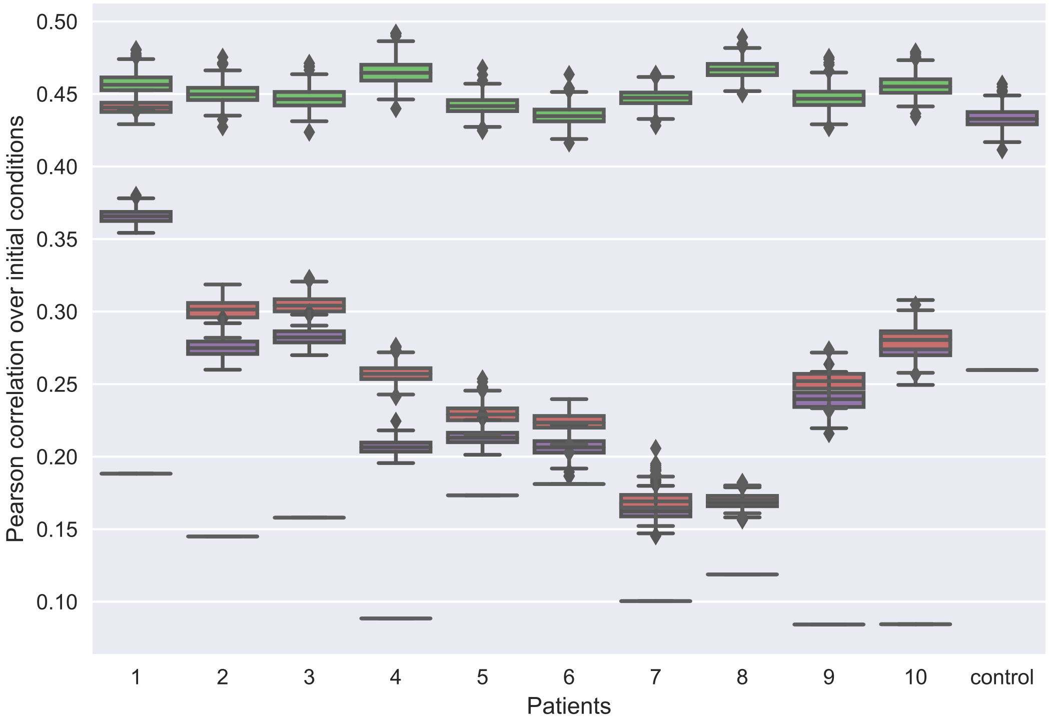

Figure S7: Optimal Pearson correlation over initial conditions for $\lambda = 40$. The purple boxplots show the Pearson correlation for the full functional connectome (without functional connectotomy). The red boxplots show the Pearson correlations after functional connectotomy for the patient-specific functional connectivity, whereas the green boxplots show the Pearson correlation after functional connectotomy for the control functional connectome. The black lines show the Pearson correlation of the average healthy structural connectome to the patient-specific and healthy control functional connectomes.

similar or is higher after functional connectotomy, both for the patient-specific and the control functional connectivity. This is sensible, as functional connectotomy excludes part of the empirical data, making the fitting less demanding for the model.
